# Mycoheterotrophic *Epirixanthes* (Polygalaceae) has a typical angiosperm mitogenome but unorthodox plastid genomes

**DOI:** 10.1093/aob/mcz114

**Published:** 2019-07-26

**Authors:** G Petersen, H Darby, V K Y Lam, H Æ Pedersen, V S F T Merckx, A Zervas, O Seberg, S W Graham

**Affiliations:** 1 Department of Ecology, Environment and Plant Sciences, Stockholm University, Stockholm, Sweden; 2 Natural History Museum of Denmark, University of Copenhagen, Copenhagen, Denmark; 3 Department of Botany, University of British Columbia, Vancouver, British Columbia, Canada; 4 UBC Botanical Garden & Centre for Plant Research, University of British Columbia, Vancouver, British Columbia, Canada; 5 Naturalis Biodiversity Centre, Leiden, The Netherlands; 6 Department of Environmental Science, Aarhus University, Denmark

**Keywords:** *Epirixanthes*, gene loss, mitochondrial genome, mycoheterotrophy, parasitic plants, plastome evolution, Polygalaceae, rearrangements, selective pressure

## Abstract

**Background and Aims:**

Fully mycoheterotrophic plants derive carbon and other nutrients from root-associated fungi and have lost the ability to photosynthesize. While mycoheterotroph plastomes are often degraded compared with green plants, the effect of this unusual symbiosis on mitochondrial genome evolution is unknown. By providing the first complete organelle genome data from Polygalaceae, one of only three eudicot families that developed mycoheterotrophy, we explore how both organellar genomes evolved after loss of photosynthesis.

**Methods:**

We sequenced and assembled four complete plastid genomes and a mitochondrial genome from species of Polygalaceae, focusing on non-photosynthetic *Epirixanthes*. We compared these genomes with those of other mycoheterotroph and parasitic plant lineages, and assessed whether organelle genes in *Epirixanthes* experienced relaxed or intensified selection compared with autotrophic relatives.

**Key Results:**

Plastomes of two species of *Epirixanthe*s have become substantially degraded compared with that of autotrophic *Polygala*. Although the lack of photosynthesis is presumably homologous in the genus, the surveyed *Epirixanthe*s species have marked differences in terms of plastome size, structural rearrangements, gene content and substitution rates. Remarkably, both apparently replaced a canonical plastid inverted repeat with large directly repeated sequences. The mitogenome of *E. elongata* incorporated a considerable number of fossilized plastid genes, by intracellular transfer from an ancestor with a less degraded plastome. Both plastid and mitochondrial genes in *E. elongata* have increased substitution rates, but the plastid genes of *E. pallida* do not. Despite this, both species have similar selection patterns operating on plastid housekeeping genes.

**Conclusions:**

Plastome evolution largely fits with patterns of gene degradation seen in other heterotrophic plants, but includes highly unusual directly duplicated regions. The causes of rate elevation in the sequenced *Epirixanthes* mitogenome and of rate differences in plastomes of related mycoheterotrophic species are not currently understood.

## INTRODUCTION

Mycoheterotrophic plants are specialized to take up carbohydrates from root-associated mycorrhizal or saprophytic fungi, thereby reducing their dependency on photosynthesis for carbon fixation. Mycoheterotrophy has evolved repeatedly in angiosperms and, while some lineages exhibit only initial stages of heterotrophy, others are fully heterotrophic and photosynthesis has been lost completely (Graham *et al.*, 2017). Most lineages of mycoheterotrophs evolved among the monocots, while in eudicots they are restricted to three families: Polygalaceae, Gentianaceae and Ericaceae (e.g. Merckx *et al.*, 2013).

A reduced dependency on photosynthesis is shared between mycoheterotrophic plants and parasitic plants, and a number of studies have described the consequential degradation of the plastid genome (plastome) in both cases (reviewed in Krause, 2011; Wicke *et al.*, 2011; Graham *et al.*, 2017; Wicke and Naumann, 2018). Heterotroph plastomes may experience structural changes, relaxed selection, elevation of substitution rates, pseudogenization or complete gene loss, and length reduction of intergenic sequence – the latter two phenomena leading to compaction of the plastome and eventually maybe even its complete loss (Wicke *et al.*, 2011, 2016; Molina *et al.*, 2014; Graham *et al.*, 2017; Wicke and Naumann, 2018). The order in which genes are lost from the plastome has been demonstrated to follow a general pattern, leading Barrett and Davis (2012) and Barrett *et al.* (2014) to propose a model for gene loss in mycoheterotrophic plants, and with only minor modifications the model has been extended to parasitic plants (Graham *et al.*, 2017). A more inclusive model accounting for several aspects of plastome changes, including structural changes and substitution rate changes, was proposed by Wicke *et al.* (2016) to apply to the evolution of non-photosynthetic plants in general.

As it is mostly responsible for cell respiration, the mitochondrial genome (mitogenome) of parasitic and mycoheterotrophic plants would not be expected to be influenced by their special lifestyle, with the exception of a possible increased substitution rate in response to a host–parasite arms race or the often small population sizes, patchy distributions and hence limited gene flow (Haraguchi and Sasaki, 1996; Bronham *et al.*, 2013). Nevertheless, massive gene loss and drastically increased substitution rates have been found in five hemiparasitic species of Viscaceae (Petersen *et al.*, 2015*a*; Skippington *et al.*, 2015; Zervas *et al.*, 2019). However, to date, no similar reductions have been discovered in other investigated clades of parasitic plants (Molina *et al.*, 2014; Bellot *et al.*, 2016; Fan *et al.*, 2016; Sanchez-Puerta *et al.*, 2017), and so the evolutionary changes occurring in Viscaceae may not be related to their parasitic lifestyle (Zervas *et al.*, 2019). One peculiarity of plant mitogenomes is a capacity to incorporate foreign DNA. Intracellular transfer of plastome DNA has been observed most frequently, but evidence is accumulating for the incorporation of DNA from other species via horizontal transfer (Sanchez-Puerta, 2014). The direct contact between parasitic plants and their hosts via haustoria is thought to facilitate horizontal transfer (Davis and Xi, 2015; Yang *et al.*, 2016), and it has even been proposed that more than half the genes in the mitogenome of the holoparasite *Lophophytum* (Balanophoraceae) are derived through such horizontal gene transfer (HGT) (Sanchez-Puerta *et al.*, 2017). The capacity of mycoheterotrophs to incorporate foreign plant DNA by HGT is unknown, and may be expected to be extremely limited based on the indirect nature of their symbiosis with green plants (nutrient transfer mediated by mycorrhizal fungi).

Plastid genome (plastome) evolution in parasitic and mycoheterotrophic plants is far better understood than mitogenome evolution. Complete plastome sequences are now available for >2500 land plants (GenBank accessed January 2019), including representatives from most clades of parasitic plants and many mycoheterotrophic lineages. However, only Ericaceae have been studied from the three eudicot families that include mycoheterotrophs (Braukmann *et al.*, 2017). Although the number of completely sequenced mitogenomes is also increasing quite rapidly (~175 in GenBank January 2019), they are harder to study because they are larger and considerably more variable in structure (e.g. Mower *et al.*, 2012). Only five complete mitogenomes have been sequenced from parasitic plants (Petersen *et al.*, 2015*a*; Skippington *et al.*, 2015; Bellot *et al.*, 2016; Fan *et al.*, 2016; Sanchez-Puerta *et al.*, 2017), and one has been sequenced for a mycoheterotrophic orchid as part of a complete genome sequencing project (Yuan *et al.*, 2018). Although data from genome skimming have provided some insights into mitochondrial evolution of parasitic plants (Zervas *et al.*, 2019), additional complete mitogenome data are badly needed for heterotrophic plants in general.

To further refine models of plastome evolution in non-photosynthetic plants, we describe the first complete plastomes from two fully mycoheterotrophic species from *Epirixanthes* (Polygalaceae). *Epirixanthes* consists of only seven species from South-east Asia (Mennes *et al.*, 2015*b*; Tsukaya *et al.*, 2016), and represents the only lineage of mycoheterotrophs in the rosid clade of eudicots. In a previously published paper, the retrievable protein-coding gene sequences from the same two species as included here were used in a phylogenetic analysis (Lam *et al.*, 2018). We also sequenced and assembled a complete plastome from a species of *Polygala*, a close photosynthetic relative of *Epirixanthes*. In addition, we assembled a complete mitogenome of *E. elongata*, the first obtained from a mycoheterotrophic plant. We used this to characterize the potential transfer of plastome genes to the mitogenome and to assess whether mycoheterotrophs are particularly prone to obtaining foreign sequences through horizontal gene transfer, either from their primary fungal host or from a secondary seed-plant host. Finally, we assess if mycoheterotrophy has had an impact on selection pressure of both plastid and mitochondrial genes.

## MATERIALS AND METHODS

### Taxon sampling

Sampling included two species of *Epirixanthes*, *E. pallida* T.Wendt and *E. elongata* Blume, and, for comparison with a closely related autotroph, *Polygala arillata* Buch.-Ham. ex D.Don was selected. Sampling of *E. elongata* included two specimens. Voucher information is listed in Table 1.

**Table 1.** Information on specimen vouchers and organellar sequences

Taxon	Voucher	No. of raw reads	Average coverage	Genome size (bp)	GenBank accession no.
*Epirixanthes elongata* Blume	Hsu 17814 (FLAS)	13 479 652	237	33,131	MK552118
*Epirixanthes elongata* Blume	Suddee *et al.* 4779 (BKF)	29 721 485*	PT: 828 MT: 637	PT: 36 275 MT: 365 168	PT: MG783393 MT: MG783394
*Epirixanthes pallida* T.Wendt	Merckx & Mennes CM001 (L)	27 608 108	323	95 420	MK552119
*Polygala arillata* Buch.-Ham. ex D.Don.	Larsen 46516 (FLAS)	9 171 790	355	164 747	MK552119

*After quality trimming.

### DNA extraction, library preparation and sequencing

DNA extraction and sequencing of *Polygala* and two specimens of *Epirixanthes* are described in Lam *et al.* (2018). For the third specimen of *Epirixanthes* (Suddee *et al.* 4779, BKF; Table 1), DNA was extracted from a silica gel-preserved sample using a DNA Plant Minikit (Qiagen) according to the manufacturer’s instructions, with the addition of a proteinase K treatment step, for 2 h at 65 °C immediately after the bead-beating step (Qiashredder). The extracted DNA was sheared using a Bioruptor (Diagenode). A 100 μL dilution at a concentration of 10 ng μL^–1^ DNA was sheared using the following conditions: five cycles of 15 s on, 90 s off. The sheared DNA was then run on a 2 % agarose gel with a 100 bp DNA ladder (Thermo Scientific) to check the size variation of the DNA fragments, which were in the range of 200–600 bp. To prepare the fragments for inserting the adaptor sequences and unique barcodes, the NEBNext DNA Sample Prep Master Mix Set 2 (New England Biolabs, Ipswich, MA, USA) was employed using blunt-end adaptors specified by Meyer and Kircher (2010). In order to determine the number of cycles needed for library construction, quantitative PCR (PCR) was performed on a 1:40 dilution of the DNA sample, using the SYBR Green Master Mix 1 (Agilent Technologies) on an Mx3500p qPCR machine (Agilent Technologies). Libraries were amplified in a total volume of 100 μL, containing 5 U of AmpliTaq Gold polymerase (Applied Biosystems, Foster City, CA, USA), 1× AmpliTaq Gold buffer, 2.5 mm MgCl_2_, 0.4 mg mL^–1^ bovine serum albumin (BSA), 0.2 mm of each dNTP, 0.2 μm IS4 forward primer, 0.2 μm indexed reverse primer and 20 μL of DNA library template. Following amplification, libraries were purified using a QIAquick PCR Purification kit (Qiagen, Hilden, Germany), according to the manufacturer’s instructions. DNA was eluted in 32 μL of EB buffer and the column was incubated for 10 min at 37 °C prior to centrifugation. The libraries were first quantified on a Qubit 2.0 (Life Technologies, Carlsbad, CA, USA) using a double-stranded DNA (dsDNA) high sensitivity assay, and then run on a TapeStation 2200 using the high sensitivity tapes (Agilent, Santa Clara, CA, USA) to determine the average insert size and molarity of each library. An Illumina Hiseq 2500 was used for 100 bp paired-end sequencing.

### Genome assembly and annotation

The plastomes of *P. arillata* and *E. pallida* were assembled and annotated previously (Lam *et al.*, 2018), although only gene sequences were used and deposited in a public repository (https://doi.org/10.6084/m9.figshare.5480608). Since only an incomplete assembly of the plastome of *E. elongata* accession Hsu 17814 was reported in the above study, we reassembled the plastome using the same procedure as for the other accession of *E. elongata*.

Raw reads of *E. elongata* were trimmed for quality, adaptors and unidentified nucleotides (Ns) using Adapter Removal (Lindgreen, 2012). The reads that passed the quality control were imported into Geneious version 10.0.5 (Biomatters Ltd). Initially the reads were mapped to the complete plastome sequences of *Cajanus cajan* (NC031429) and *Medicago truncatula* (NC003119), both Fabaceae, to roughly assess the gene content. Fabaceae is the sister group to Polygalaceae plus the poorly sampled Surianaceae. Mapping was performed using the Map to Reference option of Geneious version 10.0.5 (Biomatters Ltd) under default settings. As sequence reads only mapped to a few genes, consistent with a very small plastome, we subsequently followed a procedure based on repeated reference mapping and *de novo* assembly using unfiltered reads for assembling a complete plastome: consensus sequences calculated from the reads mapped to reference plastome genes were extracted and extended using several rounds of Map to Reference with custom settings utilizing a higher sensitivity (maximum mismatches per read 5 %; allow only 5 % gaps per read) and up to 25 iterations where unfiltered reads are automatically mapped to the consensus of the previous iteration. After each round of extension, results were inspected for errors and potential places where reads could split in two directions. Accepted new consensus sequences that did not end in a split were used for *de novo* assembly with default options of Geneious version 10.0.5 (Biomatters Ltd). For sequences that ended in a split, both alternative sequence continuations were used for subsequent rounds of extension. BLASTN analysis of the sequences against a local database of 28 complete mitogenome sequences (the database used by Petersen *et al.*, 2017, plus *Vicia faba*, KC189947) was performed to exclude potentially transferred plastid sequences (MTPTs) from assembly with true plastome sequences. This procedure was continued until all contigs were either assembled or could only be extended into a sequence already present in another contig. As the number of splits in the sequences were clearly considerably higher than the normally expected four splits [borders between the inverted repeats (IRs) and single copy (SC) regions], and regions of the sequence were suggested to be present more than twice, coverage depth was assessed to guide final manual assembly of contigs. Although the repeated sequence blocks in direct orientation could facilitate assembly into a number of smaller circles, the final complete plastome assembly presented here depicts a configuration into one circular chromosome. However, this is just one of several possible circular molecules that can be constructed. In order to validate our assembly approach, we also checked the output of a *de novo* assembly of our reads using Unicycler v. 0.4.8 beta (Wick *et al.*, 2017), which optimizes a SPAdes v. 3.13.0 (Bankevich *et al.*, 2012) assembly. The output suggested multiple possible ways to connect contigs, but did not indicate any additional sequence content for the plastome (results not shown).

To further validate the assembly, a number of PCR checks were performed. The primers used for PCR and Sanger sequencing are listed in Supplementary Data Table S1. PCR amplifications were done using Phusion High-Fidelity DNA Polymerase (Thermo Fisher Scientific, USA) and followed the general methodology in Graham and Olmstead (2000) with minor modifications: (1) initial denaturation at 98 °C for 5 min; (2) 40 cycles of the following: denaturation at 98 °C for 20 s, annealing at 60 °C for 30 s and extension at 72 °C for 2 min; and (3) final extension at 72 °C for 5 min. For cycle sequencing, we used BigDye Terminator v.3.1 (Applied Biosystems, Inc., Foster City, CA, USA) and followed the methodology in Graham and Olmstead (2000) for 25 cycles, with some modifications: (1) denaturation at 96 °C for 10 s; (2) annealing at 50 °C for 5 s; and (3) extension at 60 °C for 4 min. Sequencing reactions were run on an Applied Biosystems 3730S 48-capillary DNA analyzer (Applied Biosystems, Inc.). While positive results from PCR and sequencing confirm connections between contigs produced by *de novo* assembly, and directionality of the repeats in *E. pallida* specifically (where we focused most attention, see below), they do not preclude the presence of alternative sequence configurations, particularly in *E. elongata*, which was less extensively confirmed using PCR.

The complete mitogenome of *E. elongata* accession Suddee *et al.* 4779 was assembled using a similar procedure, despite the larger size of this genome. Initial reference mapping, which essentially we used to recover the initial gene set without regard to gene order, was based on the complete mitogenome sequences from *Millettia pinnata* (NC016742) and *V. faba* (KC189947), both Fabaceae. Thus, repeated rounds of extension and assembly were performed as above, based on the extracted gene sequences. BLASTN analyses against the assembled Polygalaceae plastome sequences were performed to detect MTPTs and avoid incorporation of genuine plastome sequences.

Both plastid and mitochondrial genes were manually annotated in Geneious version 10.0.5 (Biomatters Ltd) following comparison with Fabaceae reference organelle genomes.

### Plastome analyses

To identify structural differences, whole-plastome alignments were performed using progressiveMauve version 2.3.1 (Darling *et al.*, 2004, 2010) as implemented in Geneious version 10.0.5 (Biomatters Ltd). As a reference, we used the complete plastome sequence of *Cercis canadensis* (KF856619; Fabaceae), which has a plastome with a similar gene order to ancestral angiosperms (Schwarz *et al.*, 2015). Prior to aligning the genomes, we removed one of the two IR sequences from *C. canadensis* and *P. arillata* as well as a long direct repeat in *E. pallida*.

To test for relaxed selection of the protein-coding genes remaining in both species of *Epirixanthes* we used RELAX (Wertheim *et al.*, 2015) in the HyPhy software package (Pond *et al.*, 2005). RELAX calculates a selection intensity parameter, *k*, and tests whether selection is relaxed or intensified on a sub-set of test branches compared with a sub-set of reference branches in a pre-defined tree. In calculating *k*, relaxation is allowed to have different effects on sites subjected to purifying selection (*ω* < 1) vs. those subjected to positive selection (*ω* > 1). Relaxation will lead to changes in *ω* towards 1 (neutrality) for both categories. In the null model, selection intensity is constrained to 1 for all branches, whereas the alternative model allows *k* to differ between the reference and test branches. A likelihood ratio test (LRT) is used to reject or accept relaxed or intensified selection, but we also report values of the Akaike information criterion with a correction for finite sample size (AICc), as measures of fit of the null model and the alternative model, respectively. For comparison with other studies, we also report *ω* values calculated from the partitioned MG94xREV model. We tested 13 of 14 putative functional ribosomal genes retained in both species of *Epirixanthes* plus *matK* (putative pseudogene in *E. elongata*) in a framework that includes homologous sequences from *Polygala arillata*, *Polygala alba* and seven representatives of Fabaceae for which there are complete plastome data (taxa listed in Supplementary Data Table S2). The *rps18* gene was excluded from analysis due to alignment ambiguity and a large number of indels, but *matK* was included despite being a pseudogene in *E. elongata* due to putative pseudogenization being caused by a single deletion (see below). All sequences were aligned using MUSCLE version 3.8.24 (Edgar, 2004) as implemented in Geneious version 11.0.2 (Biomatters Ltd) either directly or to ensure that the reading frame was maintained, by applying the translation alignment option under default settings. A phylogenetic analysis of the concatenated gene alignments was performed using RAxML version 8.2.7 (Stamatakis, 2014) as implemented in Geneious version 11.0.2 (Biomatters Ltd). We used 1000 replicates of rapid bootstrapping and a GTR + GAMMA + I model. The phylogenetic tree derived from this analysis was used as input for the RELAX analyses of the individual genes, except that it was reduced to include only one representative of *E. elongata*, since RELAX does not accept identical sequences. Because of the apparently large difference in the level of plastome degradation between the two *Epirixanthes* species, we ran three sets of RELAX tests: (1) *E. elongata* as the only test branch; (2) both species of *Epirixanthes* as test branches; and (3) *Epirixanthes* plus their stem lineage as test branches. In the first two sets of analyses, the other *Epirixanthes* branches were left unlabelled, and in all analyses all remaining branches were assigned as the reference.

We also estimated synonymous (*d*_S_) and non-synonymous (*d*_N_) substitution rates by pairwise comparisons following the method of Yang and Nielson (2000) implemented as the YN00 program module in PAML version 4.9e (Yang, 2007), using the same data sets used in RELAX analyses. We ran the analysis using the graphical interface PAMLX version 1.3 (Xu and Yang, 2013).

### Mitogenome analyses

To detect regions of plastid or nuclear origin in the mitogenome of *E. elongata*, all intergenic sequences of the mitogenome were subjected to BLAST analysis. These analyses were done both using BLASTN against all GenBank sequences and locally in Geneious version 10.0.5 (Biomatters Ltd) using default settings against the assembled plastomes of *E. elongata*, *E. pallida* and *P. arillata*.

Dispersed repeats in the mitogenome were identified by blasting the complete sequence against itself using the default setting in Geneious version 10.0.5 (Biomatters Ltd). All hits with an E-value <1e^–6^ were considered.

The number of edited sites in the protein-coding genes of the mitogenome was estimated using Prep-Mt (Mower, 2009).

To detect possible cases of horizontal gene transfer of mitochondrial genes, the protein-coding genes were subjected to phylogenetic analysis. If a sequence is placed at a position other than that expected from the evolutionary origin of the species housing the sequence, horizontal gene transfer may be one (of several) explanations. After initial BLASTN analyses against all GenBank sequences, we restricted the phylogenetic analyses to include gene sequences from representatives of the fabids (the clade of eudicots including Fabales and seven other orders) for which complete mitogenome sequences were available (Supplementary Data Table S2). This taxon selection was chosen, because none of the BLASTN searches provided matches of higher similarity to sequences from other taxa. Sequences were aligned as above, and phylogenetic analyses were performed using maximum likelihood as described above.

To test for relaxed selection of the protein-coding mitochondrial genes, it was desirable to include sequences from a closer relative than the fabids with complete mitogenome sequences. Thus, we performed the tests on a sub-set of ten genes where individual gene sequences from *P. alba* and/or *P. cruciata* were available (Supplementary Data Table S2). The ten gene data sets including *Polygala* sequences were realigned (as above) and a concatenated matrix was used for phylogenetic analysis (as above). The resulting tree was used as input for RELAX analyses of individual genes. All tests were performed as above, with one branch (*E. elongata*) being the test and the remaining branches being assigned to the reference. Substitution rates were also calculated as above.

## RESULTS

### *Plastomes of* Epirixanthes *and* Polygala

**Fig. 1.**
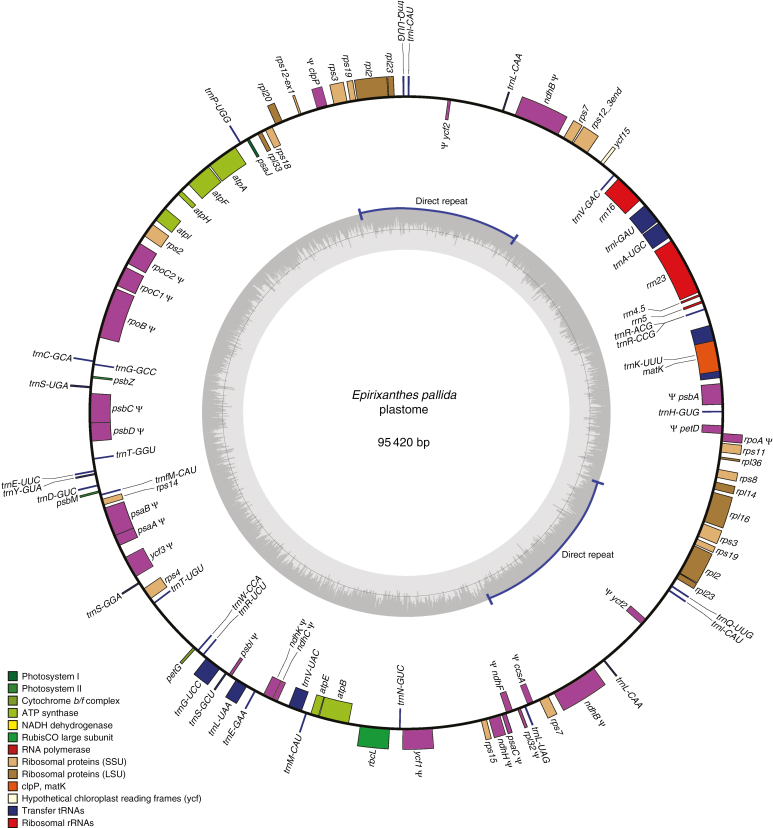
The plastome of *Epirixanthes pallida.* Direction of transcription of genes is clockwise for those on the inside and counterclockwise for those on the outside. Pseudogenes (including gene fragments) are marked by ψ. Drawing made using OGDRAW v. 1.2 (Lohse *et al.*, 2013).

**Fig. 2.**
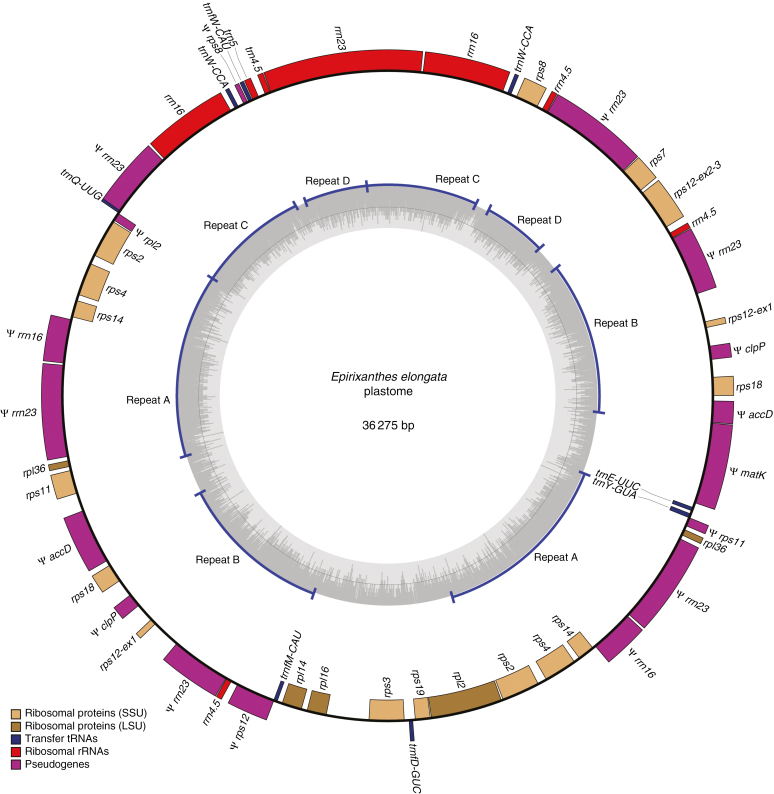
Draft plastome of *Epirixanthes elongata* (specimen Suddee *et al.* 4779, BKF). Direction of transcription of genes is clockwise for those on the inside and counterclockwise for those on the outside. Pseudogenes (including gene fragments) are marked by ψ. Four non-overlapping direct repeats are labelled A–D. Drawing made using OGDRAW v. 1.2 (Lohse *et al.*, 2013).

Information about sequencing depth for the assembled plastomes is provided in Table 1. The plastome of the autotroph *Polygala arillata* was assembled into a 164 747 bp circular chromosome (Supplementary Data Fig. S1), whereas plastomes of the mycoheterotroph species are smaller (*Epirixanthes pallida*, 95 420 bp, Fig. 1; *E. elongata*, specimen Suddee *et al.* 4779, 36 275 bp, Fig. 2; *E. elongata*, specimen Hsu 17814, 33 131 bp). This size reduction is accompanied by pseudogenization or complete loss of genes (Table 2).

**Table 2.** Summary of genes retained in the plastome of *Epirixanthes* relative to *Polygala*

Function	*Polygala arillata*	*E. pallida*	*E. elongata*	*E. elongata* MT
Photosynthesis	*psaA*, *psaB*, *psaC*, *psaI*, *psaJ*	ψ *psaA*, ψ *psaB*, ψ *psaC*, *psaJ*	—	ψ *psaA*, ψ *psaB*
	*psbA*, *psbB*, *psbC*, *psbD*, *psbE*, *psbF*, *psbH*, *psbI*	ψ *psbA*, ψ *psbC*, ψ *psbD*, ψ *psbI*	—	ψ *psbA*, ψ *psbB*, ψ *psbC*, ψ *psb*D
	*psbJ*, *psbK*, *psbL*, *psbM*, *psbN*, *psbT*, *psbZ*	*psbM*, *psbZ*		ψ *psbE*, ψ *psbF*, ψ *psbL*
	*atpA*, *atpB*, *atpE*, *atpF*, *atpH*, *atpI*	*atpA*, *atpB*, *atpE*, *atpF*, *atpH*, *atpI*	—	ψ *atpB*
	*petA*, *petB*, *petD*, *petG*, *petL*, *petN*	ψ *petD*, *petG*	—	ψ *petG*
	*ccsA*, *cemA*, *rbcL*, *ycf3*, *ycf4*	ψ *ccsA*, *rbcL*, ψ *ycf3*	—	ψ *rbcL*
	*ndhA*, *ndhB*, *ndhC*, *ndhD*, *ndhE*, *ndhF*, *ndhG*	ψ *ndhB*, ψ *ndhC*, ψ *ndhD*	—	ψ *ndhB*
	*ndhH*, *ndhI*, *ndhJ*, *ndhK*	ψ *ndhF*, ψ *ndhH*, ψ *ndhK*		
Ribosomal proteins	*rpl2*, *rpl14*, *rpl16*, *rpl20*, *rpl23*, *rpl32*, *rpl33*, *rpl36*	*rpl2*, *rpl14*, *rpl16*, *rpl20*, *rpl23*	*rpl2*, *rpl14*, *rpl16*, *rpl36*	—
		ψ *rpl32*, *rpl33*, *rpl36*		
	*rps2*, *rps3*, *rps4*, *rps7*, *rps8*, *rps11*, *rps12*, *rps14*	*rps2*, *rps3*, *rps4*, *rps7*, *rps8*, *rps11*	*rps2*, *rps3*, *rps*4, *rps7*, *rps8*, *rps11*	ψ *rps12*, ψ *rps14*
	*rps15*, *rps18*, *rps19*	*rps12*, *rps14*, *rps15*, *rps18*, *rps19*	*rps12*, *rps14*, *rps18*, *rps19*	
RNA polymerase	*rpoA*, *rpoB*, *rpoC1*, *rpoC2*	ψ *rpoA*, ψ *rpoB*, ψ *rpoC1*, ψ *rpoC2*	—	ψ *rpoB*, ψ *rpoC1*, ψ *rpoC2*
rDNAs	*rrn4.5*, *rrn5*, *rrn16*, *rrn23*	*rrn4.5*, *rrn5*, *rrn16*, *rrn23*	*rrn4.5*, *rrn5*, *rrn16*, *rrn23*	ψ *rrn4.5*, ψ *rrn*16, ψ *rrn23*
tRNAs	*trnA-UGC*, *trnC-GCA*, *trnD-GUC*	*trnA-UGC*, *trnC-GCA*, *trnD-GUC*	*trnD-GUC*, *trnE-UUC*, *trnfM-CAU*	ψ *trnA-UGC*, ψ *trnI-GAU*
	*trnE-UUC*, *trnF-GAA*, *trnfM-CAU*	*trnE-UUC*, *trnF-GAA*, *trnfM-CAU*	*trnQ-UUG*, *trnW-CCA*, *trnY-GUA*	ψ *trnL-CAA*, ψ *trnfM-CAU*
	*trnG-GCC*, *trnG-UCC*, *trnH-GUG*	*trnG-GCC*, *trnG-UCC*, *trnH-GUG*		ψ *trnfM-CAU*, *trnW-CCA*
	*trnI-CAU*, *trnI-GAU*, *trnK-UUU*	*trnI-CAU*, *trnI-GAU*, *trnK-UUU*		
	*trnL-CAA*, *trnL-UAA*, *trnL-UAG*	*trnL-CAA*, *trnL-UAA*, *trnL-UAG*		
	*trnM-CAU*, *trnN-GUU*, *trnfM-CAU*	*trnM-CAU*, *trnN-GUU*, *trnP-UGG*		
	*trnQ-UUG*, *trnR-ACG*, *trnR*-*UCU*	*trnQ-UUG*, *trnR-ACG*, *trnR-UCU*		
	*trnS-GCU*, *trnS-GGA*, *trnS-UGA*	*trnS-GCU*, *trnS-GGA*, *trnS-UGA*		
	*trnT-GGU*, *trnT-UGU*, *trnV-GAC*	*trnT-GGU*, *trnT-UGU*, *trnV-GAC*		
	*trnV-UAC*, *trnW-CCA*, *trnY-GUA*	*trnV-UAC*, *trnW-CCA*, *trnY-GUA*		
Other protein-coding genes	*accD*, ψ *clpP*, *infA*, *matK*	*accD*, ψ *clpP*, ψ *infA*, *matK*	ψ *accD*, ψ *clpP*, ψ *matK*	ψ *accD*
	*ycf1*, *ycf2*, *ycf15*	ψ *ycf1*, ψ *ycf*2, *ycf15*	—	ψ *ycf2*

Plastome genes found in the mitogenome of *E. elongata* are also listed.

A dash (—) indicates absence of all genes for that protein complex.

**Fig. 3.**
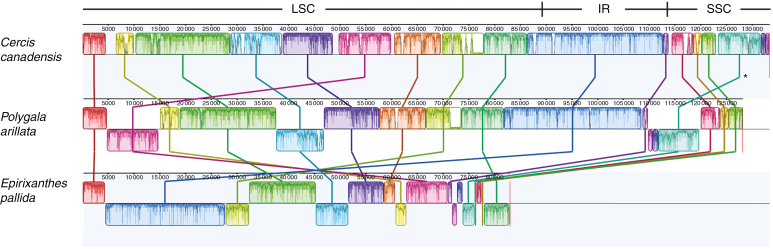
Mauve alignment showing rearrangements of the plastomes of *Polygala arillata* and *Epirixanthes pallida* relative to *Cercis canadensis*. LSC, long single copy region; SSC, small single copy region; IR, inverted repeat. The relocation and inversion marked by an asterisk * is an artefact caused by the removal of the IR-A region prior to aligning the genomes.

The Polygalaceae plastomes are all rearranged compared with the ancestral organization of angiosperms and Fabaceae (Ruhlman and Jansen, 2014; Schwarz *et al.*, 2015). In the photosynthetic member of Polygalaceae included here, *P. arillata*, approx. 9 kb of the large single copy (LSC) region that includes *petN*, *psbM*, *trnD-GUC*, *trnY-GUA*, *trnE-UUC*, *trnT-GGU*, *psbD*, *psbC*, *trnS-UGA*, *psbZ* and *trnG-GCC* has been inverted (Fig. 3; blue LSC block). Another approx. 10 kb region within the LSC including *trnL-UAA*, *trnF-GAA*, *ndhJ*, *ndhK*, *ndhC*, *trnV-UAC*, *trnM-CAU*, *atpE*, *atpB* and *rbcL* has been inverted and relocated to a position between *trnK-UUU* and *trnQ-UUG* (Fig. 3; red LSC block). A third relocation and inversion implied by the Mauve alignment (Fig. 3) is an artefact caused by the removal of the IR-A region prior to aligning the genomes and the differences between the borders of the small single copy (SSC) region and the IR of *P. arillata* and *Cercis*. The IR region of *P. arillata* is much expanded (36 534 bp) and includes the entire *ycf1* gene plus *rps15*, *ndhH*, *ndhA* and *ndhI*, which are usually part of the SSC region. Consequently, the SSC region is very short (8215 bp) and includes only *ndhF*, *rpl32*, *ccsA*, *ndhD*, *psaC*, *ndhE* and *ndhG*.

The plastome of *E. pallida* has been extensively rearranged (Fig. 1). Rearrangements include inversions and relocation, but most prominently the usual IR structure has been replaced by a direct repeat of 12 738 bp, which includes part of the standard IR region (*rps7*, *ψ ndhB*, *trnL-CAA*, ψ *ycf2*, *trnI-CAU*, *trnQ-UUG*, *rpl23* and *rpl2*), but also *rps19*, *rps3* and part of *rpl16* upstream from the usual LSC/IR-A border. We examined the *E. pallida* direct repeats in detail using PCR and Sanger sequencing reads spanning all four repeats edges of the two repeats in Fig. 1, generating both forward and reverse complement sequences that overlap these edges, to confirm continuity, and hence the directionality of the two repeats with reference to neighbouring regions. Compared with *P. arillata*, *E. pallida* has lost 22 genes completely and 25 have been pseudogenized or remain only as smaller fragments (Table 2).

The plastome of *E. elongata* is even more heavily modified, and it includes sequence regions that are duplicated up to four times (Fig. 2). The unusual number of duplications is supported by a highly variable coverage of individual genes and sequence regions (Supplementary Data Table S3) and by an equally unusual number of assembly breakpoints, some of which are located within genes (i.e. in *rpl2*, *rps8*, *rps11*, *rps12*, *rrn16* and *rrn23*). The duplicated sequences are all direct and perfect repeats, and it would be possible to construct a number of sub-circles. Figure 2 presents just one of several possible assemblies into a minimum size circular chromosome. The duplicated regions are depicted as four non-overlapping repeats (A–D) in Fig. 2, but a number of partially overlapping repeats could also be constructed. For example, repeat A contains partial *rrn16* and *rrn23* sequences that are also present in repeat C. Six regions of the plastome ranging in size from 275 to 3976 bp contain only unique sequence (Fig. 2). Read coverage of individual genes (Supplementary Data Table S3) is mostly consistent with copy numbers in the drafted plastome (Fig. 2), although high coverage of the *rrn* genes may suggest the presence of additionally repeated sequences.

The gene content of the plastome is much reduced (Table 2). Only the four ribosomal RNA genes, six tRNA genes and 14 ribosomal protein-coding genes remain. Fragments of two other protein-coding genes (*accD* and *clpP*) can be found, in addition to a 1456 bp putative pseudogene sequence of *matK*. Compared with the putative functional *matK* sequences of *E. pallida* and *P. arillata*, pseudogenization is supported by a 5 bp deletion 869 bp downstream from the start codon. Thus, the suggestion of an alternative upstream initiation codon, which would restore functionality to several previously described *matK* pseudogenes with an indel close to the conventional initiation codon (Barthet *et al.*, 2015), does not support functionality of this gene in *Epirixanthes*. The plastomes from the two specimens of *E. elongata* are largely similar, and contain the same genes. The main difference stems from specimen Hsu 17814 lacking the partial *rrn23* sequence located between *rpl36* and a partial *rrn16* sequence in specimen Suddee *et al.* 4779.

**Fig. 4.**
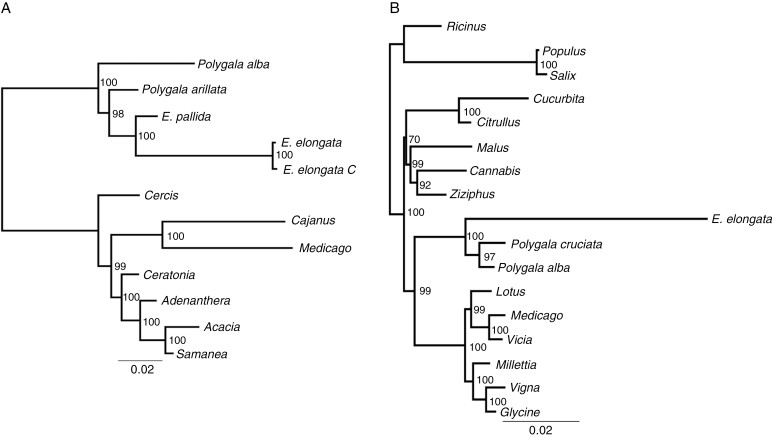
Maximum likelihood trees based on 14 plastome genes (A) and ten mitochondrial genes (B). Numbers at the nodes are bootstrap support values.

A plastid-based phylogenetic tree based on sequences from 13 ribosomal protein genes and *matK* is shown in Fig. 4A. *Epirixanthes* is monophyletic but is inferred to be embedded within a paraphyletic *Polygala*. The two species of *Epirixanthes* differ markedly in branch lengths and hence substitution rates. The higher substitution rate of *E. elongata* compared with *E. elongata* applies to nearly all 14 genes (Supplementary Data Table S4). A few of the *d*_S_ values are higher for *E. pallida* (*rps2* and *rps14*) and others are only marginally different. The tests for relaxed selection demonstrate that whether one, two or three branches of *Epirixanthes* are chosen as the test group, this has almost no statistically significant effect on the results (Supplementary Data Table S5). Only two genes, *rpl2* and *rps2*, are under significantly relaxed selection (*P* < 0.05), whereas *matK* and *rps4* show significant intensification of selection (Table 3).

**Table 3.** Test for relaxed selection of plastome genes and mitochondrial genes in *Epirixanthes*

Gene	Relaxation coefficient (*k*)*	*P*-value	Likelihood ratio	*ω* (reference)	*ω* (test)	AICc null	AICc alternative
Plastome							
***rpl2***	**0.13**	**0.0135**	**6.10**	**0.241**	**0.621**	**3905.38**	**3901.33**
*rpl14*	1.85	0.1527	2.04	0.148	0.391	2194.90	2194.98
*rpl16*	1.83	0.1697	1.89	0.140	0.304	2645.36	2645.58
*rpl36*	0.96	0.9061	0.01	0.069	0.077	682.05	684.52
***rps2***	**0**	**0.0012**	**10.47**	**0.331**	**2.157**	**4013.30**	**4004.89**
*rps3*	1.22	0.2037	1.62	0.446	0.364	4992.54	4992.99
***rps4***	**6.80**	**0.0350**	**4.45**	**0.289**	**0.576**	**3676.13**	**3673.75**
*rps7*	2.42	0.1202	2.42	0.840	0.488	2511.76	2511.44
*rps8*	1.04	0.9211	0.01	0.213	0.364	2821.64	2823.74
*rps11*	1.94	0.1868	1.74	0.360	0.325	2906.62	2906.98
*rps12*	1.23	0.9186	0.01	0.138	0.124	1824.69	1826.79
*rps14*	1.02	0.9496	0.00	0.662	0.293	2037.83	2039.98
*rps19*	0.71	0.4916	0.47	0.296	0.342	1922.13	1923.82
***matK***	**2.02**	**0.0026**	**9.09**	**0.570**	**0.634**	**12 702.78**	**12 695.72**
Mitogenome							
***atp1***	**0.53**	**0.0393**	**4.24**	**0.175**	**0.290**	**9186.19**	**9183.97**
*atp8*	0.71	0.4563	0.56	0.701	0.478	3686.60	3688.12
***cob***	**26.68**	**0.0017**	**9.87**	**0.263**	**0.431**	**5298.25**	**5290.42**
*cox1*	0.71	0.2309	1.44	0.237	0.287	7559.53	7560.12
*cox3*	1.63	0.4887	0.48	0.589	0.349	3794.12	3795.68
***matR***	**3.90**	**0.0000**	**55.24**	**0.640**	**0.626**	**12 156.58**	**12 103.36**
***nad4***	**2.05**	**0.0075**	**7.15**	**0.627**	**0.212**	**6765.64**	**6760.51**
*nad6*	0.11	0.2158	1.53	0.262	0.725	3029.27	3029.81
*nad7*	4.57	0.0878	2.91	0.497	0.573	5246.88	5246.00
***rps3***	**1.73**	**0.0054**	**7.75**	**0.699**	**0.742**	**10 805.79**	**10 800.07**

The relaxation coefficient, *k*, is the estimated selection intensity.

The values of *ω* are calculated under the partitioned MG94xREV model.

Significant results (*P* < 0.05) are in bold.

*The relaxation values apply to tests of the genus as a whole. Results from sub-set tests are provided in Supplementary Data Table S5.

### Mitogenome of Epirixanthes elongata

**Fig. 5.**
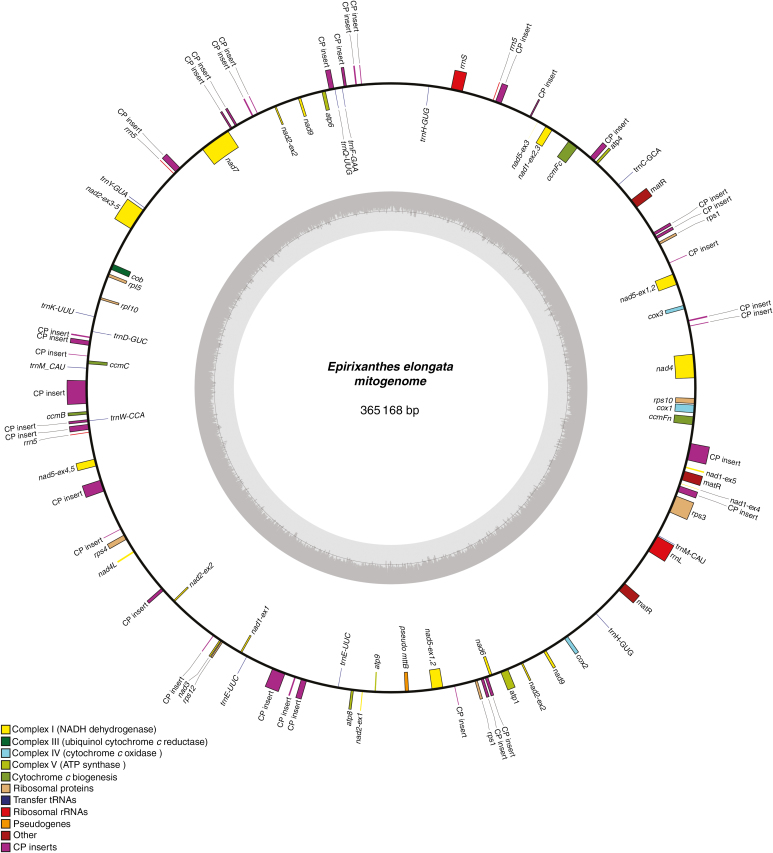
Draft mitogenome of *Epirixanthes elongata.* Direction of transcription of genes is clockwise for those on the inside and counterclockwise for those on the outside. Pseudogenes (including gene fragments) are marked by ψ. Drawing made using OGDRAW v. 1.2 (Lohse *et al.*, 2013).

**Fig. 6.**
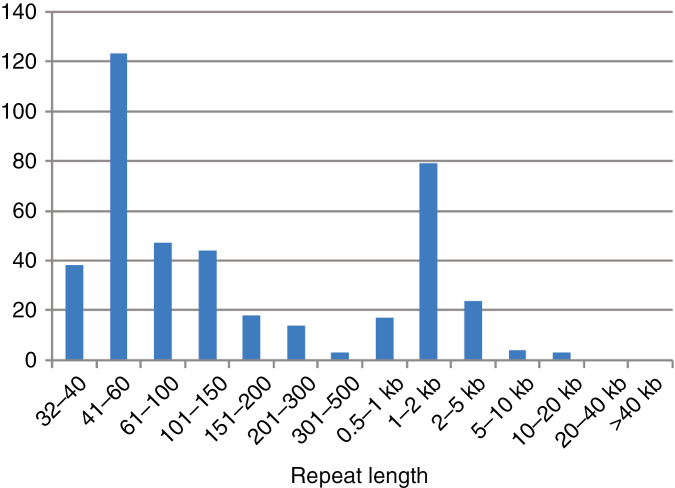
Size distribution of repeats in the mitogenome of *Epirixanthes elongata.*

The mitogenome of *E. elongata* could be assembled into a circular chromosome of 365 168 bp (Fig. 5) with an average of approx. 637× coverage from the sequence reads. The genome contains a large number of repeats (414 repeat pairs; Fig. 6; Supplementary Data Table S6) and the sequence reads reveal an abundance of breakpoints allowing for very many alternative sub-circular chromosomes to be assembled. Thus, the presented circular genome should be considered a draft master circle. The repeated sequences are not identical except for some short ones (under approx. 120 bp; Supplementary Data Table S6). The distribution of repeat length appears bimodal, with repeats either quite short (more than half are shorter than 150 bp) or within the range of 1–2 kb (Fig. 6). Approximately half (50.7 %) of the mitogenome represents unique sequence; the remaining sequence is duplicated at least once.

The gene content (Table 4; Figs 5 and 6; Supplementary Data Table S7) is similar compared with other angiosperm mitogenomes and composed of 30 protein-coding genes, ten tRNA genes and three rRNA genes. Among the core protein-coding genes, *mttB* occurs only as a pseudogene due to a premature stop codon (position 277 from the start), and *rpl16* is also represented by a pseudogene. Both pseudogenes are unequivocally supported by mapping raw reads from both specimens of *E. elongata*. Some genes are completely or partially duplicated. The completely duplicated genes are *trnE* (2×), *trnH* (2×), *nad9* (2×) and *matR* (3×). The two copies of *trnE*, *trnH* and *nad9* are identical, but the three copies of *matR* are all different. They differ in length (1812, 1818 and 1827 bp) due to a total of four 3 bp and two 6 bp indels corresponding to codons, but not by any substitutions. Potentially they are all functional. For genes with an exon–intron structure. we have indicated duplications in Fig. 5 if two copies appear equally likely to be functional, e.g. *nad5* exon1 and exon2 plus its *cis*-spliced intron occur twice, and the two copies are identical. However, *nad7*, composed of five exons and four introns that are all *cis*-spliced, is shown only once, although a long duplication includes a region spanning exon1 to part of the fourth intron (all gene fragments are annotated in the GenBank sequence).

**Table 4.** Characteristics of the *Epirixanthes elongata* mitogenome.

Feature	Value
Total size	365 168 bp
GC content	44.0 %
Protein genes	30
tRNA genes	10
rRNA genes	3
Introns (*cis*-/*trans*-spliced)	15/6
PT inserts	28 096 bp (7.7 %)
Repeats	179 947 bp (49.3 %)

A total of 21 introns are found in the mitochondrial protein-coding genes (Table 4). We observe an unexpected configuration for *nad2*, as its two first exons, which are normally separated by a *cis*-spliced intron, appear in *trans*-configuration in our master circle (Fig. 5; Supplementary Data Table S7). Although the *nad2* exon1 occurs only once, its exon2 occurs three times, with this copy number being supported by coverage data, but none of the copies is in proximity to exon1, and we find no reads to support a close connection between the two exons. The *nad4* gene is also unusual as it includes only two introns and a low number of edited sites (three) in the central exon, which is normally split by a *cis*-spliced intron in other angiosperms (Supplementary Data Tables S7 and S8). Its three putatively edited sites are located relatively close to the intron–exon borders (i.e. within a distance of 26, 44 and 116 bp, respectively) of the 938 bp long central exon. The lack of the central intron and the reduced number of edited sites also seem to be a characteristic of *E. pallida* (data not shown), but the mitochondrial read coverage was not very high for this species, rendering this interpretation less concrete. Apart from *atp6*, which completely lacked predicted RNA-edited sites, the other protein-coding genes of *E. elongata* are inferred to include edited sites (Supplementary Data Table S7).

A maximum likelihood phylogenetic tree based on sequences from the ten protein-coding genes is shown in Fig. 4B. *Epirixanthes* is placed as the sister to the two species included from *Polygala*, and the branch lengths point to an increased substitution rate in *Epirixanthes* compared with all other taxa in the tree. Increased substitution rates apply to all genes except *atp8* (Supplementary Data Table S9). However, only one gene, *atp1*, was under significantly relaxed selection (*P* < 0.05), whereas we observe significant intensification of selection in *cob*, *matR*, *nad4* and *rps3* (Table 3).

We detect 35 sequences with a length >100 bp in the mitogenome of *E. elongata* that appear to have a plastome origin (Supplementary Data Table S10). The total length of these sequences, 28 096 bp, corresponds to 7.7 % of the mitogenome and to 27 897 bp of the *E. pallida* plastome. Most of the sequences (30) have a rather low similarity (<80 %) to any other sequences available, and only one sequence (a partial *rrn23* gene) is most similar to the conspecific plastome sequence. The majority of sequences are most similar to plastome sequences of *E. pallida* or other species of Polygalaceae (mainly *Polygala* spp.). Only two are more similar to sequences from Fabaceae and Convolvulaceae, respectively, but the similarity to Polygalaceae sequences is only marginally smaller in both cases: 73.7 % to *Adenanthera* (Fabaceae) vs. 72.7 % to *Polygala*; 81.8 % to *Cuscuta* (Convolvulaceae) vs. 80.9% to *Polygala* and *E. pallida*.

## DISCUSSION

*Epirixanthes elongata* and *E. pallida* are both fully mycoheterotrophic, but their plastomes show remarkably different stages of degradation. While plastomes of both species are reduced in size, have been rearranged and have lost or pseudogenized genes, all these features are much more prominent in *E. elongata* than in *E. pallida*. According to the phylogeny of the genus, *E. elongata* is sister to the remaining species of the genus (with the possible exception of the newly described *E. confusa* Tsukaiya, M.Suleiman & H.Okada). As the genus is monophyletic (Mennes *et al.*, 2015*b*), the mycoheterotrophic mode of life in all species is most parsimoniously explained by a single loss of photosynthesis. If true, the differences in plastome degradation between *E. pallida* and *E. elongata* evolved after their divergence from their achlorophyllous most recent common ancestor, and this illustrates that different evolutionary trajectories after a common loss of photosynthesis may lead to pronounced differences in plastome reduction, similar to other clades with multiple heterotrophic taxa (e.g. Corsiaceae, Mennes *et al.*, 2015*a*; Orobanchaceae, Wicke *et al.*, 2013). Although it is less parsimonious, an alternative explanation that loss of photosynthesis and transition to mycoheterotrophy evolved independently and at different points in time along the two lineages is also worth considering.

### Structural changes of plastomes

Within Fabaceae, some major structural changes of the plastome, including loss of one IR sequence, have occurred (Schwarz *et al.*, 2015). The two major changes observed in the plastome of *P. arillata* suggest that structural changes are equally frequent even in autotrophic Polygalaceae (Fig. 3). However, following evolution of mycoheterotrophy, structural rearrangements probably increased in frequency, as described for most other parasitic and mycoheterotrophic plants (Wicke *et al.*, 2016). Loss of one of the two IR sequences is not uncommon among highly degraded plastomes (Wicke *et al.*, 2013; Lam *et al.*, 2015; Schelkunov *et al.*, 2015; Roquet *et al.*, 2016; Braukmann *et al.*, 2017; Petersen *et al.*, 2018), but the occurrence of long, direct repeats in plastomes has hitherto only been reported in some red algae (Reith and Munholland, 1993; Smith *et al.*, 2012; Wang *et al.*, 2013) and lycophytes (Mower *et al.*, 2019). However, a potential exception among angiosperms is the plastome of *Sciaphila densiflora*, where sequence coverage data suggested that some regions occur either two or four times, although they were only included once in the draft plastome (Lam *et al.*, 2015). The one 12 738 bp direct repeat observed in *E. pallida* corresponds over most of its length to part of the normal IR sequence as observed in *P. arillata*, and as such might be considered an unusual structural variant of the IR (Fig. 1; Supplementary Data Fig. S1). The pattern of several directly repeated sequences in *E. elongata*, some of which occur four times, is highly complicated, and none of the repeats has any resemblance in terms of either gene order or content to the *E. pallida* repeat. However, all the repeats include complete or partial rRNA genes normally located in the IR (Fig. 2). Due to the direct repeats it is possible to assemble an alternative plastome consisting of a number of sub-circles. The traditionally perceived circular structure of plastomes is being questioned and, although evidence suggests the existence of linear and branched–linear forms (Oldenburg and Bendich, 2015), sub-circles may be yet another possible form, comparable with the minicircles of dinoflagellates (Howe *et al.*, 2008).

The structure of the *E. elongata* plastome with its direct repeats and potential sub-circular formation has some resemblance to plant mitogenomes, and we cannot rule out that all the sequences we here assign to a circular plastome are instead integrated and duplicated in the mitogenome. The overall similarity in coverage of plastome and mitogenome sequence reads would support this scenario. However, existence of a direct repeat in the much less degraded plastome of *E. pallida* makes it likely that capacity for direct repeats is a shared characteristic of *Epirixanthes*. The identical repeated sequences thought to reside in the plastome vs. the highly divergent repeats in the mitogenome also suggest a difference in replication and/or repair mechanisms, and hence support a different genomic location.

The sequences of two IR copies are usually identical or nearly so, and IR sequences are characterized by considerably lower substitutions rates than single-copy regions (Zhu *et al.*, 2015, and references therein). In both species of *Epirixanthes*, the sequence identity of the direct repeats is maintained and, similarly to IRs in other plants, the direct repeat structure seems to preserve sequence identity. However, we do not observe any correlation between substitution rates and location of genes in single-copy vs. duplicated regions of the plastome (Supplementary Data Table S3).

### Loss and evolution of plastome genes

Based on an increasing amount of data from non-photosynthetic plants and their photosynthetic relatives, models of sequential plastome gene loss have been developed (Barrett and Davis, 2012; Barrett *et al.*, 2014; Wicke *et al.*, 2016; Graham *et al.*, 2017). The models differ slightly, but generally functional loss follows the order: (1) stress-related *ndh* genes; (2) primary photosynthesis genes (*pet*, *psa* and *psb*) and plastid-encoded polymerase genes (*rpo*); (3) other photosynthesis genes (e.g. *atp* and *rbcL*) and non-essential housekeeping genes; (4) other metabolic genes (e.g. *accD*, *clpP* and *ycf1/2*); and, finally (5) remaining housekeeping genes (*rpl*, *rps*, *rrn* and *trn*). The *trnE* gene may persist for longer than all other genes because of its dual role in translation and haem biosynthesis. The plastome of *E. elongata* fits this model perfectly, and it represents a plastome being in its final stage of degradation, with only some housekeeping genes left (Table 2). With only 14 potential functional ribosomal protein genes, four rRNA genes and six tRNA genes, it is among the most drastically reduced plastomes of mycoheterotrophic plants described to date (see Bellot and Renner, 2016; Braukmann *et al.*, 2017). The only slightly unexpected feature of the plastome is retention of a *matK* pseudogene coupled with the presence of two potentially functional genes (*rpl2* and *rps12*) requiring intron splicing, usually assumed to be provided by the *matK* gene product (Zoschke *et al.*, 2010). However, a similar situation has been observed in some other parasitic and mycoheterotrophic plants, suggesting that intron splicing may possibly occur using alternative means (see Graham *et al.*, 2017). Alternatively, pseudogenization of *matK* in *E. elongata* may be the first step towards subsequent functional loss of the genes requiring intron splicing. Putative pseudogenization/loss of function of *matK* is supported by a single indel disrupting the reading frame, which we observed in both accessions of this species examined here; this gene still appears to be under purifying selection (*ω* <1; Table 3), suggesting that pseudogenization was a recent event.

The plastome of *E. pallida* appears to be in a much earlier stage of degradation. Among primary photosynthesis genes, one or two genes within each group (*psa*, *psb* and *pet*) still remain intact (uninterrupted reading farme). Both *rbcL* and the *atp* genes are uninterrupted (Table 2), supporting their potential functionality as is hypothesized in some other heterotrophs (see Graham *et al.*, 2017). However, some metabolic genes (*clpP*, *infA* and *ycf1/2*), which accordingly are often retained at this stage, have been pseudogenized. The *clpP* gene encodes a protease subunit involved in ATP-dependent degradation of photosynthetic complexes (Peltier *et al.*, 2001), and the gene is usually only lost from highly degraded plastomes (Lam *et al.*, 2015; Bellot and Renner, 2016; Lim *et al.*, 2016; Nauman *et al.*, 2016). However, in Ericaceae, the gene is thought to have been pseudogenized prior to the evolution of mycoheterotrophy (Braukmann *et al.*, 2017), and this seems to be the case also in Polygalaceae, where we observe a pseudogene in *P. arillata*, and as in both species of *Epirixanthes*. Thus, pseudogenization of *clpP* may not be tightly correlated to mycoheterotrophy. Metabolic genes with roles outside photosynthesis and the genetic apparatus are thought to explain the long-term retention of plastid genomes; of these, only *accD* is retained in one species of *Epirixanthes* (*E. pallida*) and *trnE* in both. This is consistent with the hypothesis that these two genes are among the last non-genetic apparatus genes to be retained in plastomes of heterotrophic plants (Graham *et al.*, 2017).

In addition to size reduction, structural changes and gene loss, plastomes of non-photosynthetic plants are usually characterized by increased substitution rates, and some genes (especially but not exclusively photosynthesis genes) may experience a relaxation of selection (e.g. Barrett *et al.*, 2014; Lam *et al.*, 2015; Roquet *et al.*, 2016; Wicke *et al.*, 2016; Braukmann *et al.*, 2017; Graham *et al.*, 2017). The remaining genes with intact open reading frames in the *E. elongata* plastome follow this pattern with regards to substitution rates, although the increase in rate varies greatly among individual genes (Supplementary Data Table S4). The substitution rates of the same genes in *E. pallida* are only marginally increased or not at all (Supplementary Data Table S4), suggesting that the plastome generally evolves more slowly in this species (Fig. 4A). For both species, only two genes, *rps2* and *rpl2*, are under significantly relaxed selection, which fits a predicted pattern in which housekeeping genes are not only the last to be deleted, but also the last to experience relaxed selection. In other non-photosynthetic plants (*Sciaphila* and *Cytinus*, Ericaceae) with detailed selection data from individual genes, typically only a few of the housekeeping genes are observed to be under relaxed selection, and combined with the present evidence there is no predictable pattern concerning which genes are relaxed vs. under an intensified selective regime (Lam *et al.*, 2015; Roquet *et al.*, 2016; Braukmann *et al.*, 2017). The relaxation of some housekeeping genes may be related to initial adaptation to a parasitic lifestyle rather than the loss of photosynthesis. In Santalales, a correlation between hemiparasitism and relaxed selection of some *rps* genes has been observed (Petersen *et al.*, 2015*b*), and in Orobanchaceae a significant shift in selection pressure of the *rpl/s* genes was observed in the shift from autotrophy to parasitism, but not in the shift from hemi- to holoparasitism (Wicke *et al.*, 2016). Similar effects may be found in partially mycoheterotrophic lineages, although this has not been tested.

### Transfer of plastome genes to the mitogenome

While several genes that are now lost from the plastome of *E. elongata* have copies in its mitogenome (Table 2; Fig. 5), they are not represented there by complete reading frames. Although intracellular transfer of sequences from the plastome to the mitogenome is common among angiosperms, the transferred genes, with the possible occasional exception of tRNAs, are not thought to remain functional (Knoop, 2012; Cusimano and Wicke, 2016; Wang *et al.*, 2018).

A few of the plastome sequences inserted in the mitogenome (MTPTs) include partial rRNA gene sequences, thus corresponding to sequence still present in the *E. elongata* plastome (Supplementary Data Table S10). However, only a 1066 bp MTPT including a partial *rrn23* sequence is most similar to the conspecific plastome sequence, albeit with very low similarity (64.4 %). Two other MTPTs, which include partial *rrn23* sequences, are most similar to sequences not belonging to Polygalaceae, which might suggest either a relatively ancient transfer or an origin through horizontal transfer as proposed for MTPTs in the holoparasites *Lophophyton* (Sanchez-Puerta *et al.*, 2017) and *Pilostyles* (Bellot and Renner, 2016). Unfortunately, these MTPTs are too short to make a meaningful phylogenetic analysis. However, the similarity of the MTPTs to sequences from Fabaceae and Convolvulaceae, respectively, is only marginally higher than that to Polygalaceae sequences, and, as all three partial *rrn23* MTPTs represent non-overlapping *rrn23* sequence, we consider it likely that they stem from one transfer event of a longer sequence. This sequence could possibly have included even *rrn16*, *rrn4.5* and *trnI*, which are also present as fragmented sequences in the mitogenome (Supplementary Data Table S10). The majority of the MTPTs are most similar to plastome sequences from *E. pallida* or *Polygala*, and many inserts correspond to sequences that are located close to each other in these plastomes, e.g. *rbcL* and *atpB*. Thus, we consider it likely that the MTPTs stem from just a few transfer events of longer plastome sequences, which subsequently have degraded and become fragmented, as suggested for other angiosperms (e.g. Clifton *et al.*, 2004; Sloan and Wu, 2014; Cusimano and Wicke, 2016; Petersen *et al.*, 2017). However, it is premature to speculate on the number of transfer events, due to the rearrangements and reductions shaping the plastomes of *Epirixanthes*. There is a clear tendency towards longer MTPTs being more similar to *Polygala* plastome sequence than to sequences from *Epirixanthes*, and some MTPTs include sequence that is not currently present in any of the *Epirixanthes* plastomes (Supplementary Data Table S10). This strongly suggests that the transfer took place within Polygalaceae prior to severe reductions of the plastome, and so these represent fossilized plastid sequences that are otherwise lost from the plastomes. The lack of MTPTs with high similarity to the conspecific plastome further suggests that intracellular transfer no longer occurs. A similar scenario has been described for Orobanchaceae, where transfer of plastome sequences to both the nuclear and mitochondrial genome pre-dated plastome degradation (Cusimano and Wicke, 2016). The current sparsity of sequence data from Polygalaceae unfortunately makes attempts to date or pinpoint transfer events to particular branches through phylogenetic analysis pointless.

### Structure of the *Epirixanthes* mitogenome

The master circle representation of the *E. elongata* mitogenome (Fig. 5) should be considered to be a draft that may be artificial, as increasing evidence suggests that plant mitogenomes are mainly composed of smaller linear, circular and branched molecules (Gualberto and Newton, 2017). The abundance of repeated sequences is the basis for homologous recombination, and repeated sequences in different size classes may be involved in different types of recombination. Thus, long repeats (>500 bp) are thought to be involved in frequent homologous recombination (Gualberto and Newton, 2017), and this size class includes >100 examples in *E. elongata* (Fig. 6; Supplementary Data Table S6), suggesting a high potential for recombinational activity. Although large numbers of repeats are not uncommon, the relatively small mitogenome of *E. elongata* exhibits an unusually high frequency, and the coverage of almost half the mitogenome even exceeds the approx. 40 % repeat coverage of the large (11.3 Mb) mitogenome of *Silene conica* (Sloan *et al.*, 2012). Repeat-mediated recombination would be expected to homogenize sequence copies, but we observe that only a few short repeats are identical (none above 120 bp), whereas the longer repeats are often substantially divergent (Supplementary Data Table S6). This may possibly be caused by an increased mutation rate, as also proposed for *Silene* (Sloan *et al.*, 2012), and it is consistent with the observed increased substitution rate of the mitochondrial genes compared with the autotrophic *Polygala* (Supplementary Data Table S9).

Incomplete sequence homogenization makes it possible to retain different copies of genes, which may all be functional. The three copies of *matR* observed in *E. elongata* differ only by complete codon indels. Thus, they may all be functional and could potentially be the basis for development of an alternative functionality of the gene.

### Genes, introns and RNA editing in the *Epirixanthes* mitogenome

The gene content of the *E. elongata* mitogenome is normal compared with other angiosperms except that *mttB* is apparently pseudogenized here. To our knowledge, this is unique among angiosperms because the reported *mttB* pseudogenes in species of *Viscum* (Petersen *et al.*, 2015*a*), while highly divergent, are most probably functional genes (Skippington *et al.*, 2017). However, the massive gene loss observed in the Viscaceae (Petersen *et al.*, 2015*a*; Skippington *et al.*, 2015; Zervas *et al.*, 2019) remains restricted to this family and may not be related to parasitism. We also do not observe any evidence of HGT of the mitochondrial genes, despite suggestions that parasitic plants are particularly prone to taking up genes through the physical contact with their host (Davis and Xi, 2015, and references therein). However, the lack of direct contact between mycoheterotrophs and a plant host may strongly limit the possibility of uptake of foreign plant DNA by this means. Alternative uptake of fungal DNA could perhaps be expected in mycoheterotrophs, although HGT of fungal DNA to plants seems to be rare (Gao *et al.*, 2014).

In contrast to plant plastomes, where RNA editing affects only a few sites, plant mitochondria usually contain hundreds of predicted edited sites (Takenaka *et al.* 2013). This is the case with *E. elongata*. However, two genes have unusual patterns of editing. The *atp6* locus is predicted to not include any edited sites (Supplementary Data Table S7). Although *atp6* usually requires editing and some members of the fabids have up to 22 edited sites, the extent of editing in it is rather variable, with *Medicago* (six edited sites) and *Cannabis* (no edited sites) representing the lower range (Supplementary Data Table S8). The complete loss of editing in this gene in both *Epirixanthes* and *Cannabis* may be caused by insertion of a retrotranscriped gene replacing the original gene. This type of editing-free genes is common among angiosperms (Cuenca *et al.*, 2016, and reference therein).

The substantially low number of edited sites observed in *nad4* of *E. elongata* may also be explained by retrotransposition. The low number of edited sites is coupled with loss of nad4i976, and the lost edited sites are exactly those flanking this intron. *Epirixanthes pallida* seems to share these characteristics (data not shown), and so does a sequence of *nad4* from *P. alba*. The GenBank sequence of the latter includes a string of Ns at the location of nad4i976, but re-evaluation of the sequencing data confirms absence of an intron (R. Kellar, pers. comm.). Thus, the *nad4* gene in Polygalaceae was probably generated by recombination between a normal gene and a retrotranscribed copy in a common ancestor to *Polygala* and *Epirixanthes*. Recurrent events of loss of nad4i976 and edited sites in the Alismatales have been hypothesized to be caused by recombination between the gene and retrotranscribed copies (Cuenca *et al.*, 2016).

Another unusual intron configuration is observed in *nad2*, where nad2i156 is *trans*-spliced, although this intron has been observed in *cis*-configuration in all previously sequenced angiosperm mitogenomes. The change from *cis*- to *trans*-splicing is not correlated with any difference in the pattern of RNA editing. Although changes from *cis*- to *trans*-splicing are rare for most mitochondrial introns (Bonen, 2012), the true diversity in intron configuration among seed plants may be much wider than currently perceived. Mitochondrial genes and genomes are simply vastly underexplored.

The substitution rate of the mitochondrial genes in *E. elongata* is increased compared with *Polygala* and the other members of the fabid lineage, and three of ten genes are under significantly intensified selection (Table 3; Fig. 4B; Supplementary Data Table S9). This is consistent with the idea that a host–parasite arms race will increase evolutionary rates for the parasites and intensify the selective pressure on parasite housekeeping genes (Downton and Austin, 1995; Haraguchi and Sasaki, 1996). However, not all parasites have increased mitochondrial substitution rates and some autotroph plant lineages also exhibit profoundly increased substitution rates (e.g. Parkinson *et al.*, 2005; Bellot *et al.*, 2016; Zervas *et al.*, 2019). Thus, Zervas *et al.* (2019) demonstrated that there is no statistically significant difference in substitution rates between hemiparasites, holoparasites and autotrophic angiosperms. Our study provides the first complete mitochondrial gene complement from a mycoheterotrophic plant, so it remains to be determined whether other mycoheterotrophs also may have an increased substitution rate.

### Concluding remarks and future perspectives

Our study provides complete plastome and mitogenome sequences from a clade of mycoheterotrophic plants previously undescribed for these organellar genomes, and adds another piece of evidence to understanding evolution of non-photosynthetic plants. The plastomes of *Epirixanthes* degrade largely according to the pattern described from other lineages of non-photosynthetic mycoheterotrophic and parasitic plants. However, it is remarkable that degradation occurs at widely different speeds in the sister lineages in this genus. To understand this difference and to follow the evolution of plastomes more precisely, sequence data from the remaining species are essential. However, additional biological studies of species are also needed in order to investigate whether the differences in molecular evolution can possibly be correlated to biological factors other than lack of photosynthesis. To understand the evolution of the mitogenome, e.g. with regards to uptake of plastome sequences, obviously also requires data from additional species – not only from *Epirixanthes*, but also from other species of Polygalaceae.

While the evolution of photosynthesis genes is increasingly understood in non-photosynthetic plants, this does not apply to the housekeeping genes of both plastomes and mitogenomes, where different patterns of gene loss, substitution rates and intensity of selection are observed (Parkinson *et al.*, 2005; Lam *et al.*, 2015; Petersen *et al.*, 2015*b*; Bellot *et al.*, 2016; Roquet *et al.*, 2016; Wicke *et al.*, 2016; Braukmann *et al.*, 2017). Perhaps these patterns are truly lineage specific, but much more detailed sampling from all clades where parasitism and mycoheterotrophy have evolved is needed to discern general patterns. The evolution of organelle housekeeping genes is most probably closely linked to intracellular transfer to the nuclear genome; thus, future studies will undoubtedly benefit from technological development, further increasing sequencing capacity, allowing us to explore this genomic compartment to an extent barely possible today (Normile, 2017). While loss of photosynthesis is obviously strongly correlated with plastome evolution, the fundamental genetic basis for evolution of parasitism and mycoheterotrophy and the ongoing interaction at the genetic level between parasitic plants and their hosts should be sought in the nucleus.

## SUPPLEMENTARY DATA

Supplementary data are available online at https://academic.oup.com/aob and consist of the following. Figure S1: the plastome of *Polygala arillata.* Table S1: primers used to verify plastome assembly of *Epirixanthes pallida* and *E. elongata*. Table S2: genome and gene sequences from GenBank used for phylogenetic analyses and tests for relaxed selection. Table S3: coverage of sequence reads of *Epirixanthes elongata* plastome genes. Table S4: non-synonymous and synonymous substitution rates and *d*_N_/*d*_S_ in pairwise comparisons of plastome genes from *Polygala arillata* with other species of Polygalaceae. Table S5: test for relaxed selection of plastome genes in *Epirixanthes*. Table S6: repeated sequences in the *Epirixanthes elongata* mitogenome. Table S7: mitochondrial genes, introns and predicted edited sites in *Epirixanthes elongata.* Table S8: predicted edited sites in *atp6* and *nad4* in members of the fabids. Table S9: non-synonymous and synonymous substitution rates and *d*_N_/*d*_S_ in pairwise comparisons of mitochondrial genes from *Lotus* with species of Polygalaceae. Table S10: sequences of plastid origin in the *Epirixanthes elongata* mitogenome.

mcz114_suppl_Supplementary_Figure_S1

mcz114_suppl_Supplementary_Table_S1

mcz114_suppl_Supplementary_Table_S2

mcz114_suppl_Supplementary_Table_S3

mcz114_suppl_Supplementary_Table_S4

mcz114_suppl_Supplementary_Table_S5

mcz114_suppl_Supplementary_Table_S6

mcz114_suppl_Supplementary_Table_S7

mcz114_suppl_Supplementary_Table_S8

mcz114_suppl_Supplementary_Table_S9

mcz114_suppl_Supplementary_Table_S10

## FUNDING

This work was supported by the Danish Council for Independent Research | Natural Sciences (grant no. DFF-4002-00505) and by an NSERC Discovery Grant to S.W.G.
