# Supplementary material for: Mycoheterotrophic Epirixanthes (Polygalaceae) has a typical angiosperm mitogenome but unorthodox plastid genomes
Source: Ann Bot. 2019 Jul 26;124(5):791–807. doi: 10.1093/aob/mcz114 (PMC6868387; doi:10.1093/aob/mcz114)
Supplement: mcz114_suppl_Supplementary_Figure_S1 [file mcz114_suppl_supplementary_figure_s1.docx]

Figure S1. The plastome of *Polygala arillata.* Direction of transcription of genes is clockwise for those on the inside and counterclockwise for those on the outside. Pseudogenes are marked by ψ. Drawing made using OGDRAW v. 1.2 (Lohse et al., 2013).
