# Supplementary material for: Mycoheterotrophic Epirixanthes (Polygalaceae) has a typical angiosperm mitogenome but unorthodox plastid genomes
Source: Ann Bot. 2019 Jul 26;124(5):791–807. doi: 10.1093/aob/mcz114 (PMC6868387; doi:10.1093/aob/mcz114)
Supplement: mcz114_suppl_Supplementary_Table_S1 [file mcz114_suppl_supplementary_table_s1.docx]

Table S1. Primers used to verify plastome assembly of *Epirixanthes pallida* and *E. elongata*.

| **Primer name** | | **Primer sequence (5’ to 3’)** | | **Primer pair** |
| --- | --- | --- | --- | --- |
| Epelo_15L | | GTTCGAGTACCAGGCGCTAC | | Epelo_416R |
| Epelo_15R | | GTAGCGCCTGGTACTCGAAC | | Epelo_441L |
| Epelo_20L | | AGGCCTACGGGTCGTAAACT | | Epelo_39R |
| Epelo_21L | | TCTAGCCCCTCTGGGATGTA | | Epelo_847R |
| Epelo_21R | | GGGGAACTCGAATTTTTGGT | | Epelo_416L |
| Epelo_2828L | | TCTAAGGGTAGCCTGCTCCA | | Epelo_416L |
| Epelo_348L | | TGCACGGCTACACAGAAATC | | Epelo_416R |
| Epelo_348R | | AGGGGCTCAGGACATCTCTC | | Epelo_39L, Epelo_847L |
| Epelo_39L | | CCGTCACACTAGGGAAGCTG | | Epelo_348R, Epelo 441R |
| Epelo_39R | | CATGTCAAGCCCTGGTAAGG | | Epelo_20L, Epelo_847L98 |
| Epelo_416L | | GTGGGCGTTAGAGCATTGAT | | Epelo_2828L, Epelo_21R |
| Epelo_416R | | CCCCCATACATGGTCTTACG | | Epelo_15L |
| Epelo_441L | | GGGTGATCTATCCAGGACCA | | Epelo_15R |
| Epelo_441R | | GCTACTGGACTCTCGCCATC | | Epelo_39L |
| Epelo_847L | | TCGACGAAGACGTGTAGGTG | | Epelo_39R, Epelo_348R |
| Epelo_847R | | GATCTCGCGGATCTTTCGAT | | Epelo_21L |
| Eppal_2427F | ATCTCCCGGATAAGCCTCAC | | Eppal_2427R | |
| Eppal_2427R | TGCCCTGGCTAAACCTATTG | | Eppal_2427F | |
| Eppal_701F | TCTTGATTGGAAGGGACACC | | Eppal_701R | |
| Eppal_701R | GGGCGTTAGAGCATTGAGAG | | Eppal_701F | |
| Eppal_A1F | CATCGGTCCACACAGTTGTC | | Eppal_A1R | |
| Eppal_A1R | AGCGATGGAGTTAGCAATCG | | Eppal_A1F | |
| Eppal_B1F | TGCGTTTTGGGAGCTTCTAT | | Eppal_B2R | |
| Eppal_B2R | GCGCCTAACCCTATGAGTTG | | Eppal_B1F | |
| Eppal_C1F | GAATCCCATGAAGGACGAAA | | Eppal_C1R | |
| Eppal_C1R | ACGGGAATCCCCTTTATTTG | | Eppal_C1F | |
| Eppal_D1F | AGCATGGACCCACTCCTATG | | Eppal_D2R | |
| Eppal_D2R | CACATGGAGCCATCTCCTTA | | Eppal_D1F | |
| Eppal_E1F | TCATTCATGGGCGTTGATAA | | Eppal_E1R | |
| Eppal_E1R | CAGAGCGCAAGCTAGTGATG | | Eppal_E1F | |
| Eppal_F1F | CCGCCATCCTACCTAATGAA | | Eppal_F1R | |
| Eppal_F1R | CTCATCGCCTCGCTTTATCT | | Eppal_F1F | |
| Eppal_G1F | TTCATCGAATACGGCTTTCC | | Eppal_K1F, Eppal_G1R | |
| Eppal_G1R | AGGGGGAAGGGTTAAGGATT | | Eppal_G1F | |
| Eppal_H2F | ACGAAATCGCATTGATAGCC | | Eppal_I1F | |
| Eppal_I1F | TCAACCCACCCTTAGTACCG | | Eppal_H2F | |
| Eppal_I1R | AACTACGAGATCGCCCCTTT | | Eppal_J3R | |
| Eppal_J3R | CGTAGTTCCTACGGGGTGAA | | Eppal_I1R | |
| Eppal_K1F | GGCATGGCATCTTATGAAGG | | Eppal_G1F | |
| Eppal_L1F | TGGAACTCCAACAGGCATAA | | Eppal_L2R | |
| Eppal_L2R | GGATTCAACAAAGACGGTTCA | | Eppal_L1F | |
