# Supplementary material for: Mycoheterotrophic Epirixanthes (Polygalaceae) has a typical angiosperm mitogenome but unorthodox plastid genomes
Source: Ann Bot. 2019 Jul 26;124(5):791–807. doi: 10.1093/aob/mcz114 (PMC6868387; doi:10.1093/aob/mcz114)
Supplement: mcz114_suppl_Supplementary_Table_S2 [file mcz114_suppl_supplementary_table_s2.docx]

Table S2. Sequences from GenBank used for phylogenetic analyses and tests for relaxed selection. For the complete genomes 14 plastome genes and 10 mitogenome genes were extracted and used for RELAX analyses.

**Plastomes (Fabaceae)**

*Acacia dealbata* NC034985

*Adenanthera microsperma* NC034986

*Cajanus cajan* NC031429

*Ceratonia siliqua* KJ468096

*Cercis canadensis* KF856619

*Medicago truncatula* NC003119

*Samanea saman* KX852445

**Mitogenomes (fabids)**

Malpighiales: *Populus davidiana* NC035157

*Salix purpurea* NC029693

*Ricinus communis* NC015141

Rosales: *Cannabis sativa* NC029855

*Malus hupehensis* KR534606

*Ziziphus jujuba* NC029809

Fabales: *Glycine max* NC020455

*Lotus japonica* JN872551

*Medicago truncatula* NC029641

*Millettia pinnata* NC016742

*Vicia faba* KC189947

*Vigna radiata* HM367685

Cucurbitales *Citrullus lanatus* GQ856147

*Cucurbita pepo* GQ856148

***Polygala alba***

PT genes: KT456918 (matK), KT458144 (rpl2), KT458084 (rpl14) KT458114 (rpl16), KT458293, KT458 (rpl36), KT458631 (rps2), KT458661 (rps3), KT458691 (rps4), KT458721 (rps7), KT458751 (rps8), KT458473 (rps12), KT458503 (rps14), KT458571 (rps18), KT458601 (rps19)

MT genes: KT45880 (atp8), KT458948 (cob), KT458862 (cox1), KT458905 (cox3), KT458992 (matR), KT458036 (nad4), KT459081 (nad6), KT459126 (nad7), KT459166 (rps3)
