# Supplementary material for: Mycoheterotrophic Epirixanthes (Polygalaceae) has a typical angiosperm mitogenome but unorthodox plastid genomes
Source: Ann Bot. 2019 Jul 26;124(5):791–807. doi: 10.1093/aob/mcz114 (PMC6868387; doi:10.1093/aob/mcz114)
Supplement: mcz114_suppl_Supplementary_Table_S3 [file mcz114_suppl_supplementary_table_s3.docx]

Table S3. Coverage of sequence reads and GC content of *Epirixanthes elongata* plastome genes. If the two specimens differ, both numbers are listed (Suddee *et al*. 4779/Hsu 17814).

Gene Length GC% Copy no. Coverage

*accD* pseudogene 990/977 38.0 845.8/207.8

5’-end 600/587 38.5 1 663.9/158.7

3´-end 390 37.2 2 1127.2/281.8

*matK* pseudogene 1456 29.6 1 394.7/102.4

*rpl2* 1297/1303 796.6/186.5

5’-end 1126/1149 42.5 1 744.1/174.8

3-’end 171/154 46.2 2 1142.2/274.0

*rpl14* 369 39.8 1 723.4/168.7

*rpl16* 360/357 45.0 1 730.9/147.6

*rpl36* 114 35.1 2 1206.1/290.9

*rps2* 696 37.2 2 1382.5/346.7

*rps3* 639 36.3 1 641.5/176.5

*rps4* 603 40.3 2 1362.3/375.5

*rps7* 456 37.1 1 589.1/203.6

*rps8* 411 32.8 871.4/281.1

5’-end 328/325 31.4 1 752.1/241.8

3’-end 83/86 38.6 2 1342.7/429.7

*rps11* 426/444 43.0 881.7/175.1

5’-end 162/180 42.0 2 1162.6/201.9

3’-end 264 43.6 1 709.3/156.8

*rps12* exon1 114 43.9 2 1307.9/291.2

*rps12* exon2-3 745/751 41.9 1552.9/357.9

5’-end 711/709 42.3 2 1595.2/367.1

3’-end 34/42 32.4 1 669.5/200.6

*rps14* 312 43.3 2 1366.4/395.6

*rps18* 333/330 29.7 2 939.2/269.2

*rps19* 279 29.7 1 490.6/183.5

*rrn23* 2714/2727 55.7 3725.5/792.9

5’-end (partial)^1^ 1009/1186 54.9 4/2 3734.0/752.0^2^

3’-end (partial)^1^ 1534/1078 55.9 4 3718.5/864.2^2^

*rrn16*  1465 57.3 2938.5/807.8

5’-end 677 57.2 2 2256.3/634.5

3’-end 788 57.5 4 3524.6/958.2

*rrn4.5* 104 51.9 4 4025.5/1131.0

*rrn5* 121 54.5 1 1241.4/430.6

^1^Different fragments of *rrn23* do not have the same length. The coverage has been calculated for sequence included in all copies of 5’-end and 3’-end fragments, respectively. Thus, a central part of the gene is not included.

^2^Coverage data for Hsu 17814 does not support a factor two difference in 5’-end and 3’-end occurrence. As for Suddee *et al.* 4779 an additional repeat possibly including other *rrn* genes/fragments may exist. Potential recent transfer to the mitogenome, which has not been assembled, could also inflate coverage.
