# Supplementary material for: Mycoheterotrophic Epirixanthes (Polygalaceae) has a typical angiosperm mitogenome but unorthodox plastid genomes
Source: Ann Bot. 2019 Jul 26;124(5):791–807. doi: 10.1093/aob/mcz114 (PMC6868387; doi:10.1093/aob/mcz114)
Supplement: mcz114_suppl_Supplementary_Table_S4 [file mcz114_suppl_supplementary_table_s4.docx]

Table S4. Nonsynonymous (d_N_) and synonymous (d_S_) substitution rates and d_N_/d_S_ in pairwise comparisons of plastome genes from *Polygala arillata* with other species of Polygalaceae. P = *Polygala*, E = *Epirixanthes*, n.a. = not applicable (sequences not available for *P. alba*).

| **d_N_/d_S_** | ***matK*** | ***rpl2*** | ***rpl14*** | ***rpl16*** | ***rpl36*** | ***rps2*** | ***rps3*** | ***rps4*** | ***rps7*** | ***rps8*** | ***rps11*** | ***rps12*** | ***rps14*** | ***rps19*** |
| --- | --- | --- | --- | --- | --- | --- | --- | --- | --- | --- | --- | --- | --- | --- |
| *E. elongata* | 0.5755 | 0.4847 | 0.4156 | 0.2101 | 0.0513 | 1.9349 | 0.3986 | 0.5791 | 0.3627 | 0.2725 | 0.2397 | 0.1867 | 0.5697 | 0.5398 |
| *E. pallida* | 0.8337 | 99 | 0.1961 | 0.1915 | 0.0951 | 0.6866 | 0.3953 | 1.078 | 0.0883 | 0.3555 | 0.2621 | 0.1929 | 0.239 | 0.2228 |
| *P. alba* | 0.8821 | 2.3697 | 0.2392 | 0.3081 | n.a. | 0.3236 | 0.655 | 0.5443 | 0.5147 | 0.3028 | n.a. | 0.6271 | 1.2887 | 0.1168 |
|  |  |  |  |  |  |  |  |  |  |  |  |  |  |  |
| **d_N_** | ***matK*** | ***rpl2*** | ***rpl14*** | ***rpl16*** | ***rpl36*** | ***rps2*** | ***rps3*** | ***rps4*** | ***rps7*** | ***rps8*** | ***rps11*** | ***rps12*** | ***rps14*** | ***rps19*** |
| *E. elongata* | 0.1077 | 0.0366 | 0.0385 | 0.0306 | 0.0239 | 0.0357 | 0.066 | 0.0555 | 0.0158 | 0.0485 | 0.0567 | 0.0123 | 0.0483 | 0.0603 |
| *E. pallida* | 0.0513 | 0.0075 | 0.0179 | 0.0191 | 0.0119 | 0.0152 | 0.0299 | 0.0299 | 0.0029 | 0.0198 | 0.0195 | 0.0041 | 0.0225 | 0.0253 |
| *P. alba* | 0.0939 | 0.0132 | 0.0181 | 0.0224 | n.a. | 0.0328 | 0.0513 | 0.0288 | 0.055 | 0.0098 | n.a. | 0.0309 | 0.0679 | 0.0083 |
|  |  |  |  |  |  |  |  |  |  |  |  |  |  |  |
| **d_S_** | ***matK*** | ***rpl2*** | ***rpl14*** | ***rpl16*** | ***rpl36*** | ***rps2*** | ***rps3*** | ***rps4*** | ***rps7*** | ***rps8*** | ***rps11*** | ***rps12*** | ***rps14*** | ***rps19*** |
| *E. elongata* | 0.1872 | 0.0756 | 0.0927 | 0.1454 | 0.4079 | 0.0184 | 0.1656 | 0.0958 | 0.2232 | 0.178 | 0.2366 | 0.0658 | 0.0848 | 0.1117 |
| *E. pallida* | 0.0615 | 0 | 0.0914 | 0.0996 | 0.1256 | 0.0222 | 0.0756 | 0.0278 | 0.0323 | 0.0557 | 0.0745 | 0.0211 | 0.0942 | 0.0679 |
| *P. alba* | 0.1064 | 0.0056 | 0.0755 | 0.0728 | n.a. | 0.1012 | 0.0783 | 0.0529 | 0.1068 | 0.0325 | n.a. | 0.0493 | 0.0527 | 0.0714 |
