# Supplementary material for: Mycoheterotrophic Epirixanthes (Polygalaceae) has a typical angiosperm mitogenome but unorthodox plastid genomes
Source: Ann Bot. 2019 Jul 26;124(5):791–807. doi: 10.1093/aob/mcz114 (PMC6868387; doi:10.1093/aob/mcz114)
Supplement: mcz114_suppl_Supplementary_Table_S5 [file mcz114_suppl_supplementary_table_s5.docx]

Table S5. Test for relaxed selection of plastome genes in *Epirixanthes*.

| Gene | Relaxation coefficient (*k*) | *P*-value | Likelihood ratio (LR) | ω^a^  (Reference) | ω^a^  (Test) | AICc  null | AICc  alternative |
| --- | --- | --- | --- | --- | --- | --- | --- |
|  |  |  |  |  |  |  |  |
| *rpl2* T1^b^ | 0.19 | 0.0586 | 3.58 | 0.241 | 0.491 | 3912.53 | 3911.01 |
| T2 | 0.16 | **0.0241** | 5.09 | 0.241 | 0.566 | 3916.04 | 3913.01 |
| T3 | 0.13 | **0.0135** | 6.10 | 0.241 | 0.621 | 3905.38 | 3901.33 |
| *rpl14* T1 | 2.63 | 0.0510 | 3.81 | 0.148 | 0.607 | 2209.25 | 2207.60 |
| T2 | 1.98 | 0.1385 | 2.19 | 0.148 | 0.412 | 2207.34 | 2207.29 |
| T3 | 1.85 | 0.1527 | 2.04 | 0.148 | 0.391 | 2194.90 | 2194.98 |
| *rpl16* T1 | 2.24 | 0.1369 | 2.21 | 0.140 | 0.353 | 2658.21 | 2658.12 |
| T2 | 2.27 | 0.0651 | 3.40 | 0.141 | 0.395 | 2657.52 | 2656.24 |
| T3 | 1.83 | 0.1697 | 1.89 | 0.140 | 0.304 | 2645.36 | 2645.58 |
| *rpl36* T1 | 1.09 | 0.8575 | 0.03 | 0.069 | 0.055 | 699,42 | 701.98 |
| T2 | 1.08 | 0.8573 | 0.03 | 0.069 | 0.055 | 696.84 | 699.39 |
| T3 | 0.96 | 0.9061 | 0.01 | 0.069 | 0.077 | 682.05 | 684.52 |
| *rps2* T1 | 0 | **0.0037** | 8.43 | 0.331 | 4.511 | 4024.00 | 4017.65 |
| T2 | 0 | **0.0035** | 8.50 | 0.331 | 1.884 | 4022.20 | 4015.77 |
| T3 | 0 | **0.0012** | 10.47 | 0.331 | 2.157 | 4013.30 | 4004.89 |
| *rps3* T1 | 1.11 | 0.8125 | 0.06 | 0.446 | 0.382 | 5004.21 | 5006.23 |
| T2 | 1.11 | 0.8279 | 0.05 | 0.446 | 0.399 | 5004.27 | 5006.30 |
| T3 | 1.22 | 0.2037 | 1.62 | 0.446 | 0.364 | 4992.54 | 4992.99 |
| *rps4* T1 | 49.98 | 0.0770 | 3.13 | 0.288 | 0.402 | 3684.38 | 3683.34 |
| T2 | 39.48 | **0.0275** | 4.86 | 0.288 | 0.495 | 3686.40 | 3683.62 |
| T3 | 6.80 | **0.0350** | 4.45 | 0.289 | 0.576 | 3676.13 | 3673.75 |
| *rps7* T1 | 3.13 | 0.1202 | 2.41 | 0.837 | 0.462 | 2525.66 | 2525.36 |
| T2 | 3.14 | 0.1007 | 2.69 | 0.839 | 0.488 | 2524.33 | 2523.75 |
| T3 | 2.42 | 0.1202 | 2.42 | 0.840 | 0.488 | 2511.76 | 2511.44 |
| *rps8* T1 | 1.22 | 0.4641 | 0.54 | 0.213 | 0.273 | 2833.78 | 2835.37 |
| T2 | 1.14 | 0.6851 | 0.16 | 0.213 | 0.333 | 2833.51 | 2835.47 |
| T3 | 1.04 | 0.9211 | 0.01 | 0.213 | 0.364 | 2821.64 | 2823.74 |
| *rps11* T1 | 2.72 | 0.0568 | 3.63 | 0.360 | 0.339 | 2917.77 | 2916.26 |
| T2 | 2.11 | 0.0812 | 3.04 | 0.362 | 0.368 | 2915.73 | 2914.81 |
| T3 | 1.94 | 0.1868 | 1.74 | 0.360 | 0.325 | 2906.62 | 2906.98 |
| *rps12* T1 | 1.02 | 0.9747 | 0.00 | 0.138 | 0.124 | 1839.53 | 1841.66 |
| T2 | 1.02 | 0.9760 | 0.00 | 0.138 | 0.124 | 1837.39 | 1839.53 |
| T3 | 1.23 | 0.9186 | 0.01 | 0.138 | 0.124 | 1824.69 | 1826.79 |
| *rps14* T1 | 1.09 | 0.8530 | 0.03 | 0.663 | 0.707 | 2044.79 | 2046.94 |
| T2 | 1.03 | 0.9292 | 0.01 | 0.662 | 0.299 | 2049.71 | 2051.88 |
| T3 | 1.02 | 0.9496 | 0.00 | 0.662 | 0.293 | 2037.83 | 2039.98 |
| *rps19* T1 | 0.40 | 0.1854 | 1.75 | 0.296 | 0.556 | 1935.67 | 1936.11 |
| T2 | 0.45 | 0.2111 | 1.56 | 0.295 | 0.508 | 1933.52 | 1934.14 |
| T3 | 0.71 | 0.4916 | 0.47 | 0.296 | 0.342 | 1922.13 | 1923.82 |
| *matK* T1 | 2.25 | **0.0005** | 11.98 | 0.568 | 0.523 | 12712.06 | 12702.11 |
| T2 | 2.22 | **0.0014** | 10.21 | 0.569 | 0.605 | 12714.13 | 12705.95 |
| T3 | 2.02 | **0.0026** | 9.09 | 0.570 | 0.634 | 12702.78 | 12695.72 |

^a^Calculated under the Partitioned MG94xREV model.

^b^T1: one test branch (*E. elongata*); T2: two test branches (*E. elongata*+*E. pallida*); T3: three test branches (*E. elongat*a+*E. pallida*+ancestral branch).

Significant results (P<0.05) in bold.
