# Supplementary material for: Mycoheterotrophic Epirixanthes (Polygalaceae) has a typical angiosperm mitogenome but unorthodox plastid genomes
Source: Ann Bot. 2019 Jul 26;124(5):791–807. doi: 10.1093/aob/mcz114 (PMC6868387; doi:10.1093/aob/mcz114)
Supplement: mcz114_suppl_Supplementary_Table_S6 [file mcz114_suppl_supplementary_table_s6.docx]

Table S6. Repeated sequences in the *Epirixanthes elongata* mitogenome.

Repeat A Repeat B

Similarity % Length Start End Start End e-value

99.481 15033 16272 31243 277832 292847 0.0

99.482 11975 78780 90710 321977 310008 0.0

99.373 11327 104660 115935 309024 297708 0.0

99.427 7859 130652 138484 64738 72589 0.0

99.157 7830 71110 78900 228232 236043 0.0

98.631 6718 136912 143578 132151 125469 0.0

98.059 5203 8667 13855 114468 119596 0.0

99.363 4870 35657 40515 321777 326629 0.0

98.910 4495 284936 289399 211733 207256 0.0

98.888 4495 23352 27814 211733 207256 0.0

99.414 4267 115209 119465 94506 98767 0.0

98.604 4298 9441 13724 94506 98767 0.0

97.752 3603 346226 349768 35679 39260 0.0

97.775 3595 321806 325374 346226 349768 0.0

98.573 3433 160852 164263 47957 44552 0.0

98.522 3382 44475 47818 305265 308634 0.0

99.361 3288 160991 164263 105050 108332 0.0

98.626 3348 226396 229727 301036 297708 0.0

98.226 3382 105050 108409 47818 44475 0.0

99.060 3298 160991 164263 308634 305342 0.0

97.795 3401 226396 229773 112621 115982 0.0

99.131 2533 33282 35798 354540 357066 0.0

98.412 2582 261505 264050 247508 244932 0.0

98.603 2505 132133 134622 78778 81263 0.0

98.283 2505 66224 68708 78778 81263 0.0

98.121 2501 319493 321977 68708 66226 0.0

97.885 2506 132135 134622 321977 319493 0.0

99.635 2193 151510 153699 49942 52129 0.0

98.072 2230 117656 119861 254283 256493 0.0

98.676 2115 301999 304103 294069 296168 0.0

98.440 2115 109566 111664 296168 294069 0.0

97.971 1971 254283 256228 11900 13855 0.0

99.353 1856 166486 168340 363318 365168 0.0

98.497 1863 166518 168367 10230 8378 0.0

98.688 1829 8412 10230 365168 363350 0.0

99.492 1771 271917 273682 8336 10102 0.0

97.601 1834 96958 98767 254283 256097 0.0

98.598 1783 169336 171117 130560 132325 0.0

98.957 1726 166650 168367 273682 271959 0.0

97.411 1777 271945 273721 66437 64699 0.0

99.116 1697 271988 273682 365168 363482 0.0

96.466 1811 130586 132351 10166 8364 0.0

97.227 1767 166582 168340 169362 171100 0.0

96.758 1789 166582 168367 130586 132337 0.0

96.946 1768 169362 171100 363414 365168 0.0

96.319 1793 169362 171117 10166 8390 0.0

97.353 1738 130652 132351 273682 271945 0.0

96.761 1760 363414 365168 130586 132308 0.0

96.959 1743 8364 10102 66437 64738 0.0

96.524 1755 297462 299189 130324 132054 0.0

97.818 1696 64738 66411 169428 171117 0.0

97.267 1720 271971 273682 171117 169428 0.0

97.747 1687 64738 66394 363482 365168 0.0

97.037 1721 166650 168367 64738 66423 0.0

95.147 1772 137005 138746 299189 297462 0.0

96.294 1646 363380 365011 72623 71013 0.0

96.294 1646 71013 72623 168178 166548 0.0

96.058 1649 71013 72623 8570 10200 0.0

98.444 1542 8667 10200 228232 229761 0.0

96.154 1612 136912 138484 365011 363414 0.0

96.092 1612 166582 168178 138484 136912 0.0

96.658 1586 190757 192320 168057 166495 0.0

97.610 1548 363380 364914 229761 228232 0.0

97.545 1548 228232 229761 168082 166548 0.0

96.595 1586 363327 364889 192320 190757 0.0

95.913 1615 136912 138484 8570 10166 0.0

95.291 1635 136912 138510 170938 169336 0.0

96.857 1559 190757 192297 8692 10230 0.0

95.875 1600 71013 72589 132151 130586 0.0

97.693 1517 228259 229761 190763 192267 0.0

95.522 1608 169362 170938 72589 71013 0.0

97.439 1523 169336 170842 297682 299189 0.0

97.495 1517 190757 192267 71135 72623 0.0

95.997 1574 363350 364914 115995 114468 0.0

97.673 1504 297708 299189 10166 8667 0.0

95.870 1574 166518 168082 115995 114468 0.0

97.782 1488 293810 295280 220289 221761 0.0

97.152 1510 166582 168082 297708 299189 0.0

96.186 1547 272150 273682 71013 72521 0.0

96.239 1542 190763 192297 114495 115995 0.0

97.020 1510 363414 364914 297708 299189 0.0

95.992 1547 272150 273682 136912 138416 0.0

96.054 1546 71110 72635 114468 115982 0.0

96.948 1507 228232 229727 170842 169362 0.0

96.217 1533 64738 66242 72521 71013 0.0

98.338 1444 272247 273682 228232 229663 0.0

97.235 1483 137030 138484 190757 192233 0.0

95.893 1534 136912 138416 66242 64738 0.0

96.301 1514 137005 138484 228232 229727 0.0

98.125 1440 297770 299189 273682 272247 0.0

98.054 1439 197935 199360 77901 76478 0.0

98.054 1439 233616 235039 199360 197935 0.0

96.526 1497 169362 170842 115935 114468 0.0

96.462 1498 114468 115935 137005 138484 0.0

96.450 1493 130586 132054 115935 114468 0.0

96.218 1507 130586 132054 229727 228232 0.0

95.759 1509 297708 299189 72589 71110 0.0

97.759 1428 64738 66145 297770 299189 0.0

96.169 1488 297708 299162 192233 190763 0.0

96.169 1488 169362 170817 192233 190765 0.0

96.875 1440 272247 273682 114468 115873 0.0

96.875 1440 64738 66145 229663 228232 0.0

96.855 1431 272272 273682 190757 192169 0.0

96.779 1428 64738 66145 115873 114468 0.0

95.296 1488 190765 192233 132029 130586 0.0

95.502 1423 64738 66118 192169 190763 0.0

98.852 1219 301999 303210 220548 221761 0.0

98.277 1219 110459 111664 221761 220548 0.0

99.637 1101 280379 281479 174926 176023 0.0

98.728 1101 18805 19895 174926 176023 0.0

98.966 967 268598 269554 217630 218596 0.0

99.020 816 220499 221309 331850 332665 0.0

97.672 816 331850 332665 294020 294818 0.0

98.957 767 301999 302758 331899 332665 0.0

97.118 798 191500 192297 94506 95287 0.0

98.305 767 110911 111664 332665 331899 0.0

97.100 793 363350 364138 95287 94506 0.0

97.100 793 166518 167306 95287 94506 0.0

97.055 781 228994 229773 94506 95274 0.0

96.903 775 71866 72635 94506 95274 0.0

98.487 727 297708 298434 95227 94506 0.0

98.356 730 169362 170091 95227 94506 0.0

98.753 722 176573 177289 148529 147812 0.0

97.521 726 130586 131303 95227 94506 0.0

96.982 729 94506 95227 137760 138484 0.0

97.748 666 273021 273682 94506 95165 0.0

97.879 660 64738 65389 95165 94506 0.0

98.457 324 195500 195818 38928 39251 8.05e-159

97.256 328 325038 325365 195500 195818 1.46e-155

95.732 328 195500 195818 349437 349759 1.66e-148

97.758 223 271945 272167 78996 78779 5.44e-104

97.196 214 199087 199295 310736 310524 6.20e-97

93.860 228 78779 78996 8587 8364 3.21e-94

97.073 205 89996 90194 76747 76543 4.77e-92

97.073 205 89996 90194 233885 233681 4.77e-92

95.327 214 310524 310736 76543 76747 5.81e-91

95.327 214 310524 310736 233681 233885 5.81e-91

95.215 209 199087 199295 89996 90194 3.01e-88

96.465 198 78778 78970 170921 171117 1.28e-86

92.857 210 168161 168367 78778 78982 9.84e-82

89.640 222 271945 272166 321763 321977 7.56e-77

93.923 181 364993 365168 78778 78953 4.77e-73

93.750 176 35651 35816 168373 168198 2.47e-70

86.784 227 321763 321977 8364 8586 8.63e-70

87.678 211 35657 35855 271959 272166 3.01e-69

91.525 177 364995 365168 35855 35679 6.64e-65

94.304 158 35657 35807 66423 66266 8.08e-64

92.638 163 132175 132337 35807 35657 9.85e-63

92.638 163 78820 78982 35807 35657 9.85e-63

96.599 147 170958 171104 35816 35675 9.85e-63

91.018 167 346201 346367 168365 168208 4.19e-61

94.702 151 321789 321934 171117 170967 4.19e-61

93.038 158 356919 357066 168373 168216 1.46e-60

83.333 216 8378 8586 35657 35855 1.78e-59

93.960 149 356925 357066 66423 66275 6.22e-59

93.960 149 356925 357066 321777 321925 6.22e-59

92.208 154 356925 357066 78982 78829 7.57e-58

92.208 154 356925 357066 132337 132184 7.57e-58

92.208 154 356925 357066 271959 272112 7.57e-58

98.450 129 365040 365168 321934 321806 2.64e-57

98.425 127 235917 236043 272167 272041 3.22e-56

90.184 163 321777 321934 168367 168207 1.12e-55

90.968 155 346226 346379 78953 78808 3.92e-55

90.968 155 346226 346379 132308 132163 3.92e-55

97.656 128 364993 365120 235916 236043 3.92e-55

100.000 120 356947 357066 365168 365049 3.92e-55

92.958 142 346226 346367 171100 170968 1.37e-54

92.958 142 346226 346367 271988 272120 1.37e-54

97.656 128 235916 236043 168161 168287 4.78e-54

97.656 128 235916 236043 170921 171047 4.78e-54

96.850 127 235917 236043 8587 8465 5.82e-53

96.124 129 356943 357066 171104 170976 5.82e-53

90.476 147 346221 346367 8407 8540 2.48e-51

95.312 128 235916 236043 66224 66346 2.48e-51

100.000 111 61462 61572 12610 12500 3.02e-50

100.000 111 61462 61572 97660 97550 3.02e-50

100.000 111 61462 61572 118358 118248 3.02e-50

100.000 111 61462 61572 254992 254882 3.02e-50

94.531 128 235916 236043 132133 132255 1.05e-49

89.437 142 346226 346367 365168 365041 3.67e-49

86.164 159 356925 357066 8378 8532 4.48e-48

86.452 155 66254 66394 346379 346226 5.45e-47

88.806 134 356947 357066 346226 346359 8.09e-45

97.938 97 249288 249384 334417 334513 6.22e-40

88.976 127 310581 310695 35437 35311 9.23e-38

87.597 129 235918 236043 35855 35727 9.23e-38

98.901 91 360537 360627 110550 110640 9.23e-38

98.901 91 360537 360627 221670 221580 9.23e-38

98.901 91 360537 360627 295184 295094 9.23e-38

98.901 91 360537 360627 303119 303029 9.23e-38

87.302 126 321854 321977 236043 235918 1.67e-34

89.344 122 356585 356705 310695 310581 5.83e-34

87.705 122 199128 199237 356585 356705 2.48e-32

85.827 127 233734 233844 35437 35311 8.65e-32

85.827 127 76596 76706 35437 35311 8.65e-32

75.207 242 259970 260191 199006 199241 8.65e-32

93.069 101 199141 199237 35338 35437 3.02e-31

85.586 111 101631 101725 90262 90152 3.02e-31

96.250 80 165526 165605 83867 83788 4.48e-29

96.250 80 316889 316968 165526 165605 4.48e-29

96.250 80 346288 346367 236043 235964 4.48e-29

100.000 71 72620 72690 26778 26848 1.56e-28

100.000 71 229758 229828 26778 26848 1.56e-28

94.253 87 330631 330715 75363 75448 1.56e-28

100.000 71 229758 229828 208296 208226 1.56e-28

100.000 71 72620 72690 208296 208226 1.56e-28

94.253 87 330631 330715 232501 232586 1.56e-28

100.000 71 72620 72690 288359 288429 1.56e-28

100.000 71 229758 229828 288359 288429 1.56e-28

86.066 122 356585 356705 76706 76596 5.46e-28

98.667 75 332983 333056 189619 189693 5.46e-28

86.066 122 233734 233844 356705 356585 5.46e-28

86.066 122 76596 76706 356705 356585 5.46e-28

81.897 116 310456 310571 101631 101725 1.90e-27

88.679 106 259970 260072 76828 76725 6.65e-27

84.426 122 356585 356705 90037 90142 6.65e-27

88.679 106 259970 260072 233966 233863 6.65e-27

97.222 72 356995 357066 236043 235972 2.32e-26

89.109 101 35338 35437 90050 90142 2.83e-25

98.507 67 259994 260060 23238 23304 9.87e-25

98.507 67 259994 260060 284822 284888 9.87e-25

100.000 59 336741 336799 59419 59477 5.11e-22

90.909 77 339527 339596 164319 164243 1.78e-21

100.000 58 199451 199508 199382 199439 1.78e-21

91.667 72 240820 240885 101431 101360 6.23e-21

98.333 60 35378 35437 260128 260187 6.23e-21

98.333 60 356646 356705 260128 260187 6.23e-21

94.030 67 233875 233940 23304 23238 7.58e-20

94.030 67 199032 199097 23238 23304 7.58e-20

94.030 67 76737 76802 23304 23238 7.58e-20

94.030 67 284822 284888 76802 76737 7.58e-20

94.030 67 284822 284888 199032 199097 7.58e-20

94.030 67 284822 284888 233940 233875 7.58e-20

100.000 51 362027 362077 226444 226394 1.13e-17

93.939 66 151481 151543 17448 17383 3.93e-17

93.939 66 278953 279018 151543 151481 3.93e-17

100.000 50 35841 35890 213035 213084 3.93e-17

100.000 50 321963 322012 213035 213084 3.93e-17

100.000 50 346365 346414 213035 213084 3.93e-17

100.000 49 362027 362075 112669 112621 1.37e-16

100.000 49 362027 362075 300988 301036 1.37e-16

96.226 53 286424 286476 13406 13354 4.79e-16

96.226 53 24837 24889 13406 13354 4.79e-16

96.226 53 119101 119153 24889 24837 4.79e-16

96.226 53 98403 98455 24889 24837 4.79e-16

96.226 53 286424 286476 98455 98403 4.79e-16

96.226 53 286424 286476 119153 119101 4.79e-16

100.000 47 341296 341342 21712 21666 1.67e-15

98.077 52 76596 76647 66421 66471 1.67e-15

98.077 52 233734 233785 66421 66471 1.67e-15

98.077 52 90091 90142 66471 66421 1.67e-15

98.077 52 321729 321779 76647 76596 1.67e-15

98.077 52 78980 79030 76596 76647 1.67e-15

98.077 52 233734 233785 78980 79030 1.67e-15

98.077 52 90091 90142 79030 78980 1.67e-15

98.077 52 321729 321779 90091 90142 1.67e-15

96.154 52 213032 213083 92695 92746 1.67e-15

98.077 52 76596 76647 132335 132385 1.67e-15

98.077 52 233734 233785 132335 132385 1.67e-15

98.077 52 90091 90142 132385 132335 1.67e-15

98.077 52 321729 321779 233785 233734 1.67e-15

100.000 47 341296 341342 283296 283250 1.67e-15

97.959 49 219018 219066 10476 10428 5.83e-15

97.959 49 219018 219066 95533 95485 5.83e-15

97.959 49 219018 219066 116241 116193 5.83e-15

90.909 66 132317 132378 35455 35390 2.04e-14

90.909 66 78962 79023 35455 35390 2.04e-14

90.909 66 66403 66464 35455 35390 2.04e-14

90.909 66 321736 321797 35390 35455 2.04e-14

90.909 66 356658 356723 66464 66403 2.04e-14

90.909 66 356658 356723 79023 78962 2.04e-14

90.909 66 356658 356723 132378 132317 2.04e-14

100.000 45 288096 288140 154661 154617 2.04e-14

100.000 45 208518 208562 154617 154661 2.04e-14

100.000 45 26515 26559 154661 154617 2.04e-14

97.917 48 270985 271032 270693 270740 2.04e-14

90.909 66 356658 356723 321736 321797 2.04e-14

88.710 62 128033 128094 21416 21477 7.10e-14

88.710 62 140965 141026 21477 21416 7.10e-14

95.918 49 92698 92746 35841 35889 7.10e-14

95.918 49 35841 35889 92698 92746 7.10e-14

95.918 49 321963 322011 92698 92746 7.10e-14

95.918 49 346365 346413 92698 92746 7.10e-14

88.710 62 283000 283061 128033 128094 7.10e-14

88.710 62 283000 283061 141026 140965 7.10e-14

100.000 44 132335 132378 199237 199194 7.10e-14

100.000 44 78980 79023 199237 199194 7.10e-14

100.000 44 66421 66464 199237 199194 7.10e-14

100.000 44 321736 321779 199194 199237 7.10e-14

100.000 44 321736 321779 310624 310581 7.10e-14

100.000 44 66421 66464 310581 310624 7.10e-14

100.000 44 78980 79023 310581 310624 7.10e-14

100.000 44 132335 132378 310581 310624 7.10e-14

95.918 49 92698 92746 321963 322011 7.10e-14

95.918 49 92698 92746 346365 346413 7.10e-14

92.727 55 155112 155166 147676 147624 2.48e-13

95.833 48 210196 210243 255732 255779 2.48e-13

74.468 141 259915 260046 90009 90141 8.65e-13

100.000 42 146767 146808 146704 146745 8.65e-13

92.308 52 336354 336405 269963 270014 8.65e-13

89.655 58 349600 349657 4887 4831 3.02e-12

89.655 58 325206 325263 4887 4831 3.02e-12

89.655 58 195668 195725 4887 4831 3.02e-12

89.655 58 39096 39153 4887 4831 3.02e-12

95.833 48 46523 46570 57858 57903 3.02e-12

95.833 48 162230 162277 57903 57858 3.02e-12

95.833 48 106304 106351 57903 57858 3.02e-12

95.833 48 307328 307375 57858 57903 3.02e-12

97.727 44 299439 299482 59006 58963 3.02e-12

97.727 44 114175 114218 58963 59006 3.02e-12

97.727 44 227939 227982 58963 59006 3.02e-12

97.727 44 342318 342361 128076 128033 3.02e-12

97.727 44 342318 342361 140983 141026 3.02e-12

73.611 144 259915 260046 199100 199236 3.02e-12

79.570 93 35346 35436 259956 260046 1.05e-11

79.570 93 356614 356704 259956 260046 1.05e-11

100.000 40 287492 287531 351717 351678 1.05e-11

100.000 40 209132 209171 351678 351717 1.05e-11

100.000 40 25905 25944 351717 351678 1.05e-11

95.455 44 160966 161009 9320 9277 3.68e-11

95.455 44 364259 364302 47843 47800 3.68e-11

95.455 44 9277 9320 47800 47843 3.68e-11

95.455 44 298555 298598 47843 47800 3.68e-11

95.455 44 115050 115093 47800 47843 3.68e-11

95.455 44 170208 170251 47843 47800 3.68e-11

95.455 44 167427 167470 47843 47800 3.68e-11

95.455 44 228830 228873 47800 47843 3.68e-11

95.455 44 131420 131463 47843 47800 3.68e-11

95.455 44 272857 272900 47800 47843 3.68e-11

95.455 44 65506 65549 47843 47800 3.68e-11

97.727 44 76543 76586 101683 101725 3.68e-11

97.727 44 233681 233724 101683 101725 3.68e-11

95.455 44 65506 65549 160966 161009 3.68e-11

95.455 44 272857 272900 161009 160966 3.68e-11

95.455 44 131420 131463 160966 161009 3.68e-11

95.455 44 228830 228873 161009 160966 3.68e-11

95.455 44 167427 167470 160966 161009 3.68e-11

95.455 44 170208 170251 160966 161009 3.68e-11

95.455 44 115050 115093 161009 160966 3.68e-11

95.455 44 298555 298598 160966 161009 3.68e-11

95.455 44 364259 364302 160966 161009 3.68e-11

100.000 39 47215 47253 201613 201651 3.68e-11

100.000 39 161543 161581 201651 201613 3.68e-11

100.000 39 105612 105650 201651 201613 3.68e-11

100.000 39 308029 308067 201613 201651 3.68e-11

89.474 57 233685 233739 35549 35494 1.28e-10

89.474 57 76547 76601 35549 35494 1.28e-10

90.741 54 35494 35546 90137 90188 1.28e-10

90.741 54 356762 356814 90137 90188 1.28e-10

83.333 66 282998 283063 150460 150395 1.28e-10

83.333 66 21414 21479 150460 150395 1.28e-10

97.561 41 186193 186233 224733 224693 1.28e-10

87.500 64 310577 310636 260191 260128 1.28e-10

89.474 57 233685 233739 356817 356762 1.28e-10

89.474 57 76547 76601 356817 356762 1.28e-10

95.455 44 310545 310586 35537 35494 4.48e-10

95.455 44 199232 199273 35494 35537 4.48e-10

97.500 40 71705 71744 47800 47839 4.48e-10

97.500 40 137599 137638 47800 47839 4.48e-10

97.500 40 191335 191374 47800 47839 4.48e-10

97.500 40 191335 191374 161009 160970 4.48e-10

97.500 40 137599 137638 161009 160970 4.48e-10

97.500 40 71705 71744 161009 160970 4.48e-10

72.603 146 233735 233872 260046 259915 4.48e-10

72.603 146 76597 76734 260046 259915 4.48e-10

95.455 44 310545 310586 356805 356762 4.48e-10

95.455 44 199232 199273 356762 356805 4.48e-10

100.000 36 2363 2398 125998 125963 1.56e-09

100.000 36 2363 2398 143056 143091 1.56e-09

95.122 41 340644 340684 239126 239166 1.56e-09

71.812 149 259915 260046 310723 310582 1.56e-09

100.000 35 35421 35455 8364 8398 5.46e-09

100.000 35 356689 356723 8364 8398 5.46e-09

86.792 53 13354 13406 210196 210243 5.46e-09

86.792 53 98403 98455 210196 210243 5.46e-09

86.792 53 119101 119153 210196 210243 5.46e-09

86.792 53 286424 286476 255779 255732 5.46e-09

86.792 53 24837 24889 255779 255732 5.46e-09

100.000 35 35421 35455 271945 271979 5.46e-09

100.000 35 356689 356723 271945 271979 5.46e-09

97.436 39 36747 36784 90702 90740 1.91e-08

97.436 39 322869 322906 90702 90740 1.91e-08

97.436 39 347274 347311 90702 90740 1.91e-08

100.000 34 1 34 346225 346192 1.91e-08

86.792 53 23184 23236 76796 76745 6.65e-08

86.792 53 23184 23236 199038 199089 6.65e-08

86.792 53 23184 23236 233934 233883 6.65e-08

86.792 53 233883 233934 284820 284768 6.65e-08

86.792 53 199038 199089 284768 284820 6.65e-08

86.792 53 76745 76796 284820 284768 6.65e-08

100.000 33 152787 152819 328261 328229 6.65e-08

100.000 33 51214 51246 328261 328229 6.65e-08

100.000 32 222102 222133 93992 94023 2.32e-07

100.000 32 220954 220985 128895 128926 2.32e-07

100.000 32 111229 111260 128926 128895 2.32e-07

100.000 32 294468 294499 128895 128926 2.32e-07

100.000 32 302403 302434 128895 128926 2.32e-07

100.000 32 332310 332341 128895 128926 2.32e-07

100.000 32 332310 332341 140171 140140 2.32e-07

100.000 32 302403 302434 140171 140140 2.32e-07

100.000 32 294468 294499 140171 140140 2.32e-07

100.000 32 111229 111260 140140 140171 2.32e-07

100.000 32 220954 220985 140171 140140 2.32e-07

100.000 32 267554 267585 186981 187012 2.32e-07

87.755 49 101683 101725 199295 199247 2.32e-07

87.755 49 233873 233921 260201 260154 2.32e-07

87.755 49 199051 199099 260154 260201 2.32e-07

87.755 49 76735 76783 260201 260154 2.32e-07

84.211 57 336790 336846 10363 10415 8.11e-07

84.211 57 336790 336846 95420 95472 8.11e-07

84.211 57 336790 336846 116128 116180 8.11e-07

81.356 59 150398 150456 128093 128035 8.11e-07

81.356 59 150398 150456 140966 141024 8.11e-07

94.737 38 196818 196855 178577 178612 8.11e-07
