# Supplementary material for: Mycoheterotrophic Epirixanthes (Polygalaceae) has a typical angiosperm mitogenome but unorthodox plastid genomes
Source: Ann Bot. 2019 Jul 26;124(5):791–807. doi: 10.1093/aob/mcz114 (PMC6868387; doi:10.1093/aob/mcz114)
Supplement: mcz114_suppl_Supplementary_Table_S7 [file mcz114_suppl_supplementary_table_s7.docx]

Table S7. Mitochondrial protein coding genes, introns and predicted edited sites in *Epirixanthes elongata.*

| **Gene** | **Predicted number of edited sites** | **Introns**  ***cis* *trans*** | |
| --- | --- | --- | --- |
| *atp1* | 3 |  |  |
| *atp4* | 11 |  |  |
| *atp6* | 0 |  |  |
| *atp8* | 4 |  |  |
| *atp9* | 4 |  |  |
| *ccmB* | 30 |  |  |
| *ccmC* | 28 |  |  |
| *ccmFc* | 22 | ccmFci829 |  |
| *ccmFn* | 28 |  |  |
| *cob* | 14 |  |  |
| *cox1* | 16 |  |  |
| *cox2* | 13 |  |  |
| *cox3* | 6 |  |  |
| *matR* | 11-12 |  |  |
| *nad1* | 17 | nad1i477, nad1i728 | nad1i394, nad1i669 |
| *nad2* | 26 | nad2i709, nad2i1282 | nad2i156, nad2i542 |
| *nad3* | 7 |  |  |
| *nad4* | 20 (exon1:14, exon2:3, exon3:3) | nad4i461, nad4i1399 |  |
| *nad4L* | 14 |  |  |
| *nad5* | 25 | nad5i230, nad5i1872 | nad5i1455, nad5i1477 |
| *nad6* | 10 |  |  |
| *nad7* | 27 | nad7i140, nad7i209, nad7i676, nad7i917 |  |
| *nad9* | 7 |  |  |
| *rpl5* | 11 |  |  |
| *rpl10* | 4 |  |  |
| *rps1* | 3-4 |  |  |
| *rps3* | 9 | rps3i74 |  |
| *rps4* | 16 |  |  |
| *rps10* | 5 | rps10i235 |  |
| *rps12* | 6 |  |  |
