# Supplementary material for: Mycoheterotrophic Epirixanthes (Polygalaceae) has a typical angiosperm mitogenome but unorthodox plastid genomes
Source: Ann Bot. 2019 Jul 26;124(5):791–807. doi: 10.1093/aob/mcz114 (PMC6868387; doi:10.1093/aob/mcz114)
Supplement: mcz114_suppl_Supplementary_Table_S8 [file mcz114_suppl_supplementary_table_s8.docx]

Table S8: Predicted edited sites in *atp6* and *nad4* in members of the fabids.

Taxa *atp6 nad4*(edited sites per exon)

Malpighiales: *Populus davidiana* 11 38: 17-7-10-4

*Salix purpurea* 11 37: 17-7-10-3

*Ricinus communis* 11 34: 16-7-9-2

Rosales: C*annabis sativa* 0 43: 17-8-14-4

*Malus hupehensis* 20 39: 18-6-12-3

*Ziziphus jujuba* 22 46: 18-9-15-4

Fabales: *Glycine max* 17 41: 16-8-13-4

*Lotus japonica* 20 42: 17-8-13-4

*Medicago truncatula* 6 42: 17-8-13-4

*Millettia pinnata* 17 41: 16-8-13-4

*Polygala alba* 19: 13-1-2-3

*Vicia faba* 10 ?: 16-?-13-4

*Vigna radiata* 18 41: 16-8-13-4

Cucurbitales: *Citrullus lanatus* 22 38: 19-5-11-3

*Cucurbita pepo* 22 33: 16-5-9-3
