# Supplementary material for: Mycoheterotrophic Epirixanthes (Polygalaceae) has a typical angiosperm mitogenome but unorthodox plastid genomes
Source: Ann Bot. 2019 Jul 26;124(5):791–807. doi: 10.1093/aob/mcz114 (PMC6868387; doi:10.1093/aob/mcz114)
Supplement: mcz114_suppl_Supplementary_Table_S9 [file mcz114_suppl_supplementary_table_s9.docx]

Table S9. Nonsynonymous (d_N_) and synonymous (d_S_) substitution rates and d_N_/d_S_ in pairwise comparisons of mitochondrial genes from *Lotus* with species of Polygalaceae

| **d_N_/d_S_** | ***atp1*** | ***atp8*** | ***cob*** | ***cox1*** | ***cox3*** | ***matR*** | ***nad4*** | ***nad6*** | ***nad7*** | ***rps3*** |
| --- | --- | --- | --- | --- | --- | --- | --- | --- | --- | --- |
| *Epirixanthes* | 0.0114 | 1.1464 | 0.3866 | 0.2498 | 0.4427 | 0.6651 | 0.5528 | 0.5886 | 0.4677 | 0.6964 |
| *Polygala* | 0.0666 | 1.5138 | 0.3874 | 0.2316 | 0.7844 | 0.4344 | 1.0444 | 0.2658 | 0.2843 | 0.4801 |
|  |  |  |  |  |  |  |  |  |  |  |
| **d_N_** | ***atp1*** | ***atp8*** | ***cob*** | ***cox1*** | ***cox3*** | ***matR*** | ***nad4*** | ***nad6*** | ***nad7*** | ***rps3*** |
| *Epirixanthes* | 0.0133 | 0.1084 | 0.0257 | 0.0226 | 0.0281 | 0.1791 | 0.0469 | 0.0506 | 0.0319 | 0.1111 |
| *Polygala* | 0.0053 | 0.1370 | 0.0159 | 0.0128 | 0.0227 | 0.0236 | 0.0403 | 0.0173 | 0.0116 | 0.0353 |
|  |  |  |  |  |  |  |  |  |  |  |
| **d_S_** | ***atp1*** | ***atp8*** | ***cob*** | ***cox1*** | ***cox3*** | ***matR*** | ***nad4*** | ***nad6*** | ***nad7*** | ***rps3*** |
| *Epirixanthes* | 0.1193 | 0.0946 | 0.0664 | 0.0906 | 0.0634 | 0.2693 | 0.0849 | 0.0860 | 0.0681 | 0.1595 |
| *Polygala* | 0.0792 | 0.0905 | 0.0412 | 0.0553 | 0.0289 | 0.0543 | 0.0386 | 0.0651 | 0.0408 | 0.0736 |
