# Supplementary material for: Mycoheterotrophic Epirixanthes (Polygalaceae) has a typical angiosperm mitogenome but unorthodox plastid genomes
Source: Ann Bot. 2019 Jul 26;124(5):791–807. doi: 10.1093/aob/mcz114 (PMC6868387; doi:10.1093/aob/mcz114)
Supplement: mcz114_suppl_Supplementary_Table_S10 [file mcz114_suppl_supplementary_table_s10.docx]

Table S10. Sequences of plastid origin (MTPTs) in the *Epirixanthes elongata* mitogenome.

| **Insert coding sequence** | **Similarity** | **Position in MT** | **Length/MT** | **Position in Pa PT** | **Length/Pa PT** |
| --- | --- | --- | --- | --- | --- |
| PT atpB | 86.8% Ep | 11,785-11,922 | 138 | 7,645-7,772 | 128 |
| PT rrn16 | 68.1% Ep | 12,758-13,029 | 272 | 100,112-100,403 | 292 |
| PT rrn4.5 | 71.1% Ep | 24,453-24,566 | 114 | 107,162-107,301 | 140 |
| PT trnI | 68.3% Ep | 29,658-30,225 | 568 | 102,097-102,738 | 642 |
| PT psbL+F+E | 73.6% Pa | 30,487-31,083 | 597 | 64,427-65,027 | 601 |
| PT rbcL | 75.9% Ep | 48,897-49,935 | 1,039 | 5,578-6,613 | 1036 |
| PT psbA | 76.5% Ep | 63,529-63,867 | 339 | 327-694 | 368 |
| PT psaA | 66.9% Pa | 69,788-70,980 | 1,193 | 50,077-51,366 | 1290 |
| PT atpB | 86.8% Ep | 96,843-96,980 | 138 | 7,645-7,772 | 128 |
| PT rrn16 | 68.1% Pc | 97,808-98,078 | 271 | 100,112-100,403 | 292 |
| PT rpoC2 | 75.9% S | 99,756-100,323 | 568 | 26,221-26,750 | 530 |
| PT rps12 exon1 | 73.9% Ep | 102,193-103,187 | 995 | 69,620-70,671 | 1052 |
| PT atpB | 86.8% Ep | 117,541-117,678 | 138 | 7,645-7,772 | 128 |
| PT rrn16 | 67.7% Ep | 118,506-118,776 | 271 | 100,112-100,403 | 292 |
| PT rrn23+trnA | 73.7% A | 122,026-122,498 | 473 | 103,720-104,255 | 536 |
| PT ycf2 | 73.2% Pa | 123,146-123,554 | 409 | 91,402-91,845 | 444 |
| PT psaA | 66.9% Pa | 135,687-136,879 | 1,193 | 50,077-51,366 | 1290 |
| PT trnL | 80.6% Pa | 172,800-173,054 | 255 | 93,585-93,857 | 273 |
| PT ndhB | 76.8% Pa | 173,589-174,513 | 925 | 95,239-96,208 | 970 |
| PT rrn23 | 81.8% C | 176,461-176,564 | 104 | 106,247-106,347 | 101 |
| PT rpoC1/rpoC2 | 68.1% Pa | 181,180-185,628 | 4,449 | 27,539-32,799 | 5261 |
| PT petG, trnW, trnP | 76.9% Pa | 188,613-189,124 | 512 | 66,378-66,890 | 513 |
| PT rrn23 | 64.4% Ee | 189,517-190,583 | 1,066 | 104,531-105,848 | 1318 |
| PT psbC/D | 70.8% Pa | 200,430-202,700 | 2,271 | 39,250-41,849 | 2600 |
| PT rrn4.5 | 71.1% Ep | 210,519-210,632 | 114 | 107,162-107,301 | 140 |
| PT ndhB | 74.0% Pa | 224,276-224,937 | 662 | 94,612-95,236 | 625 |
| PT IGS | 68.4% Ep | 237,660-237,805 | 146 | 93,373-93,547 | 175 |
| PT psaB+rps14+trnfM | 70.2% Pa | 250,650-253,281 | 2,632 | 46,919-49,797 | 2879 |
| PT rrn16 | 68.1% Ep | 255,145-255,416 | 272 | 100,112-100,403 | 292 |
| PT accD | 74.8% Ep | 256,486-257,603 | 1,118 | 58,021-59,185 | 1165 |
| PT rrn4.5 | 71.1% Ep | 286,040-286,153 | 114 | 107,162-107,301 | 140 |
| PT trnI | 68.3% Ep | 291,254-291,821 | 568 | 102,097-102,738 | 642 |
| PT psbL+F+E | 73.5% Pa | 292,088-292,687 | 600 | 64,427-65,027 | 601 |
| PT psbB | 71.5% Pa | 344,946-346,155 | 1,210 | 71,318-72,684 | 1367 |
| PT rpoC1+ rpoB | 72.9% Pa | 351,281-354,542 | 3,262 | 33,028-36,618 | 3591 |

Ee = *Epirixanthes elongata*, Ep = *Epirixanthes pallida*, Pa = *Polygala arillata*, Pc = *Polygala cruciata*, S = *Securidaca diversifolia* (Polygalaceae), A = *Adenanthera microsperma* (Fabaceae), C = *Cuscuta* spp. (Convolvulaceae).
